# Supplementary material for: Silkworm cultivation predating the Silk Road in southern Central Asia (2000 BCE)
Source: Sci Adv. 2026 Jul 23;12(30):eaec8738. doi: 10.1126/sciadv.aec8738 (PMC13394462; doi:10.1126/sciadv.aec8738)
Supplement: Supplementary file 1 — Supplementary Text Figs. S1 to S13 Tables S1 to S4 References [file sciadv.aec8738_sm.pdf]

Science Advances
AAAS

Supplementary Materials for

Silkworm cultivation predating the Silk Road in southern Central Asia (2000 BCE)

Xinying Zhou *et al.*

Corresponding author: Xinying Zhou, zhouxinying@ivpp.ac.cn; Qingyou Xia, xiaqy@swu.edu.cn; Xinyi Liu, liuxinyi@wustl.edu

Sci. Adv. **12**, eaec8738 (2026)
DOI: 10.1126/sciadv.aec8738

This PDF file includes:

Supplementary Text
Figs. S1 to S13
Tables S1 to S4
References

Supplementary Text

Research into pre-Han silks/silk-like technology

The silk trade profoundly influenced Old World politics and commerce, promoting communication between countries in early historical periods. It is now widely accepted that *B. mori* evolved domestication traits and genetically diverged from its wild ancestor *B. mandarina* in China (*32*). Archaeological discoveries based on both proteomics and morphological analyses have identified Neolithic silk or silk-like fibers across the Yellow and Yangtze River regions (*4*). The earliest evidence of prehistoric silk fibroin came from the Jiahu site, through the analysis of soil samples collected from three 8,500-year-old tombs using mass spectrometry and proteomics (*2*). In the Xiyincun site (ca. 5000–3000 BCE), an "artificially cut" cocoon of what was reported to be *Bombyx mori* was discovered in 1926 (*59–61*); however, a follow-up analysis suggested that it is *Ronditia menaciana* (*60*). Nonetheless, questions still remain regarding this cocoon, warranting further identification and investigation into the reasons why it was cut. The earliest physical silk remains thus far reported have been recovered from the Qingtai site (ca. 3500 BCE) in central China, but they have only been morphologically assigned as *Bombyx* silk ((*62*). In the Yangtze River region, silk remains have been reported from the Qianshanyang site (ca. 2750 BCE) in 1958 (*63*, *64*). The site was recently re-excavated but no additional silk fibers were recovered and the date of this site was reassessed to 2200–2000 BCE (*4*, *65*).

Whereas silk, itself, rarely preserves, artistic depictions of the caterpillars have also been widely recognized at Neolithic sites across eastern China, such as at Hemudu (ca. 5000 BCE)(*66*), Xiwangcun (Yangshao context)(*67*), and Shicun (ca. 4000 BCE)(*68*). Meanwhile, the earliest textual evidence of silk as an economic fiber is found among the Shang Dynasty (1600–1046 BCE) oracle-bone inscriptions(*69*).

Beyond eastern China, recent research from the Quman cemetery (ca. 500 BCE) on the Pamir Plateau, Xinjiang, also reports findings of wool and *Bombyx* silk (*70*). An additional heavily cited ancient example comes from the dress of a frozen Pazyryk burial from the Altai, affectionately named the Ice Madden (ca. 400 BCE). The textile has been identified as silk, but there have been contradictory claims over the origin of the silk, and some scholars have suggested that it is a wild silk whereas others have identified it as Tussar silk from India (*71*).

In South Asia, sericulture traditions utilizing other silkmoth species, including Eri silk (*Samia cynthia ricini*), Muga silk (*Antheraea assamensis*), and Tussar silk (*Antheraea* spp.), are

distinct from the East Asian tradition. These insects consume the leaves of a variety of trees, including castor bean (*Richinus communis*), som (*Machilus gamblei*), soalu (*Litsea monopetala*), oak (*Quercus* spp.), arjun (*Terminalia arjuna*), and sal (*Shorea robusta*) (*10*). In the Indus Civilization (2800–1500 BCE), microscopic analysis of archaeological thread fragments found inside bronze and chlorite beads from two important Indus sites, Harappan and Chanudaro (2600-2200 BCE) have yielded silk fibers (*11*). Specifically, there are two distinct thread forms in the samples from Harappan, and they appear to be from two different species of silkmoth in *Antheraea* sp., and the silk from Chanudaro is of another (yet unidentified) species, possibly an Eri silk (*Philosamia* spp.) (*11*). From the slightly later site of Nevasa (1500–1050 BCE) in modern day India, a single thread from a bead has been reported as silk, while the nature of processing was not determinable (*72*).

In Europe and West Asia, many claims of ancient "silk" have been made, based on the methods of morphological observation and/or amino acid compositional analysis. Additionally, it has been proposed that a separate Mediterranean origin of silk production was based on wild moths of *Pachypasa otus* in the Lasiocampidae family, a possible explanation of claims of early silk in Greece (*12*, *13*). For instance, a calcified cocoon of *Pachypasa otus* was reported from excavations at Thera, which had been destroyed and buried by a volcanic eruption in the mid second millennium BCE (*73*). Other claims for pre-Han "silk" remains come from Gordion (ca. 700 BCE)(*14*), Sardis (ca. 600–500 BCE)(*14*, *74*), Kerameikos (ca. 500 BCE)(*14*, *75–77*), Hohmichele (ca. 500 BCE) (*14*, *77–82*), Hochdorf (ca. 530 BCE)(*14*, *82*, *83*), and Altrie (450–25 BCE) (*14*, *84*, *85*). It is essential to emphasize that nearly all of these claims have been either disproven or challenged, and in several cases cannot be confirmed due to a loss of the material (*15*, *16*). There is only one report of "silk" from Africa, namely Deir al Medina (ca. 1000 BCE), in which a thread was confidently thought to be from a wild silk (*14*); however, later examination has thrown serious doubt on this find (*13*). We see this complex history of claims for silk being proposed and then refuted as providing further impetus for our present study.

Due to the perishability of silk, it is rarely found archaeologically, leaving open questions about the possibility of additional centers and whether connections may have existed across Asia prior to the Han Dynasty, likely via cultural diffusion. Meanwhile, the search for ancient silk has produced a complex research history of proposed claims that have later been refuted. While morphological methods are useful, distortion (delamination of fibers) due to decomposition can

make them problematic, and early attempts at molecular methods, notably amino acid compositional analysis, have, in multiple cases, provided contradictory results(*15*). Regarding the status of the study of prehistoric silk, Bender Jørgensen referred to it as "an unhappy situation, particularly since the arguments are based on highly specialized scientific analyses that require specialized knowledge to interpret" (*15*).

Research into the BMAC

The Bactria-Margiana Archaeological Complex (BMAC), a culturally connected entity that flourished between around 2500–1500 BCE, and experienced rapid growth during the period from 2200 to 1800 BCE. Its distribution extended eastward to the Pamirs, westward to Turkey, northward to Uzbekistan, and southward to Afghanistan, with the Amu Darya River basin as its core area (*54*). It has long been recognized that this social and demographic phenomenon represented the rise of a culturally unified region of proto-urban polities, with the presence of palaces, temples, and cemeteries with ornamented scepters, wine cups of silver and gold, figurines, and uninscribed seals (*53*, *86*). While, debate continues over their political unification, whether a functioning state or a conglomerate of city-states, increasing research is illustrating the economic development and connection through broader exchange networks (*53–55*, *86*, *87*).

Owing to its strategic location at the heart of Eurasia, the polities in southern Central Asia became wrapped into a broad commerce network, across a variety of geographic and cultural horizons. Archaeologists most often discuss connections between the Indus Valley, the Iranian Plateau, Mesopotamia, the Persian Gulf, the northern steppe zone, and western China (*55*, *88*). Long-distance translocation of commodities, materials or ideas could be achieved through several mechanism including direct contact and exchange, sustained transfer via markets, or cultural learning and inspirations. At the time, some of the earliest evidence for pack animals in Central Asia, in the form of oxcarts and camels provide further indications of the growing network of exchange (*89*, *90*). The presence of Indus- related discoveries from Shortughai, Dashly (*91*, *92*), Djarkutan (*93*), and especially Gonur Tepe, including a number of ivory items, etched carnelian beads or faience bangles, stone seated small sculptures (*88*, *94–96*), an inscribed Indus seal (*97*), and glazed faience figurines of monkeys (*98*, *99*), confirmed tight relations between the two areas.

Archaeobotanical evidence has also shown that a complex agricultural system was maintained across the Margiana region, broadly, which incorporated a more diverse array of

crops than had been cultivated together at any time prior in Central Eurasia (*100*). Between ca. 1700–1500 BCE, an aridification of the environment is often considered to be the main explanation for the abandonment or reduction in densities of populations that archaeologists demarcate as the terminus of the BMAC, while other factors have been proposed and the cultural changes were likely multifactorial (*55*).

Provenance

The materials presented in this study include silkworm cocoons, wood charcoal and plant seeds. These samples were collected during a joint excavation project between the Institute of Vertebrate Paleontology and Paleoanthropology (IVPP), Chinese Academy of Sciences, and the National Center of Archeology, Academy of Sciences of the Republic of Uzbekistan in 2017. The project was led by Xinying Zhou and Jianxin Wang from China, and Mutalibjon Khasannov and Akhmadali A. Askarov from Uzbekistan. It involved a small-scale excavation at Sapalli Tepe, and sample collection from Djarkutan and Molleli in southern Uzbekistan. The samples were obtained from these three sites using the floatation method during the joint excavation.

The three silkworm cocoons were analyzed by Zhiqing Li and Qingyou Xia from Southwest China through morphological observations and protein profiling analysis. All wood charcoal fragments were identified by Hui Shen from IVPP, based on the anatomical features observed under scanning electron microscopy. Xinying Zhou and Guanhan Chen from IVPP, and Robert Spengler from Max Planck Institute of Geoanthropology conducted the identification of plant seeds based on morphological traits. Radiocarbon dating of the samples was performed by Beta Analytic in Miami, USA, using accelerator mass spectrometry (AMS) measurements. All materials are currently housed at IVPP, and the morphological features and statistical data are reported in this study.

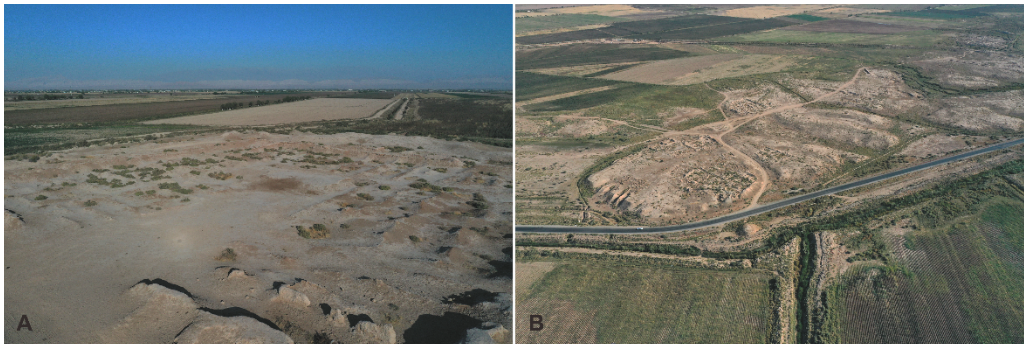

Fig. S1. The overview of Sapalli Tepe (A) and Djarkutan (B).

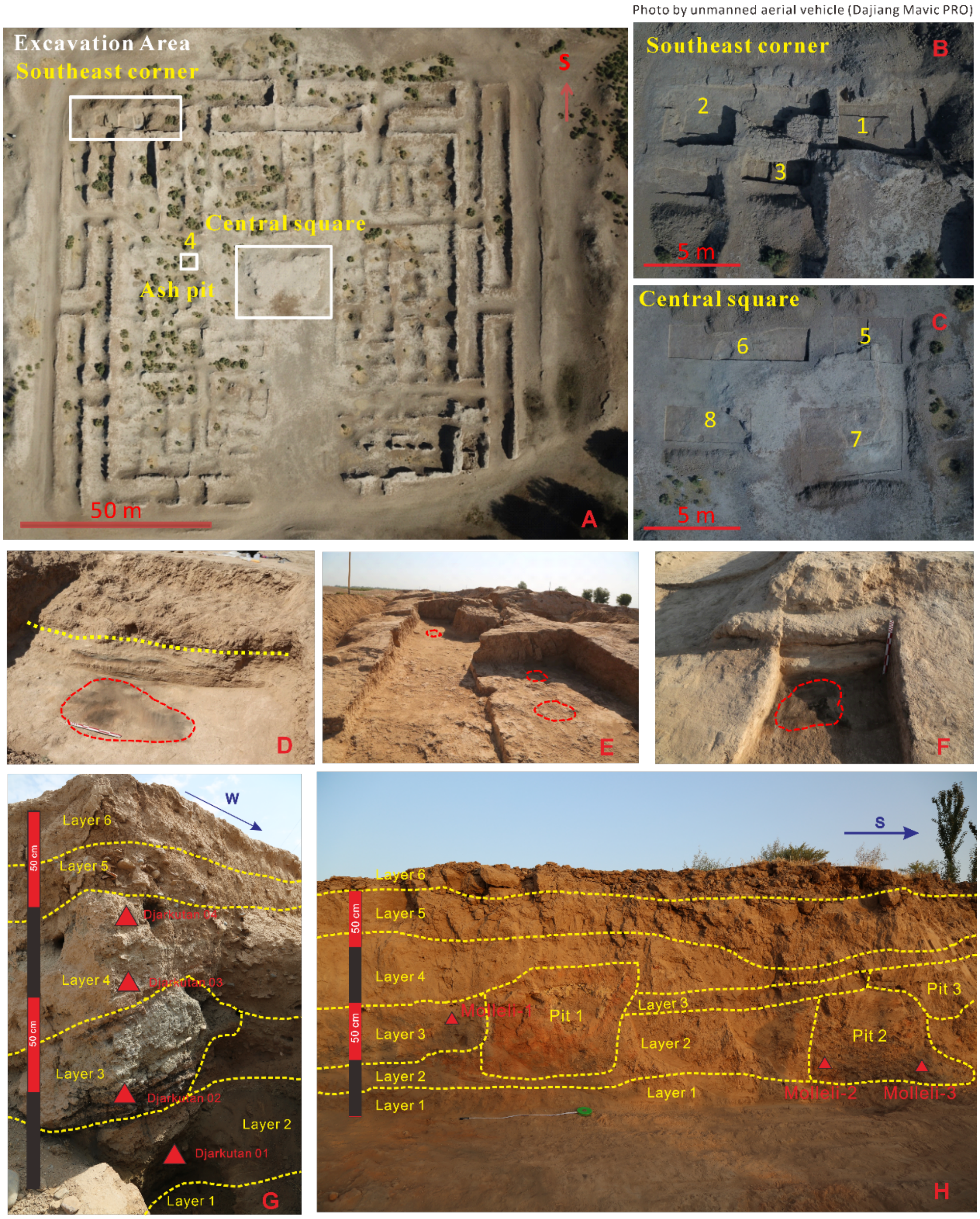

Fig. S2. The study sections and sampling layer of Sapalli Tepe, Djarkutan, Molleli and sample locations. (A) The sample collection location at Sapalli Tepe. (B) Location of trench in excavation area 1 of Sapalli Tepe. (C) Location trenches 5-8 in excavation area 2 of Sapalli Tepe. (D-F) Ash pit location in the trenches 1-3 at Sapalli Tepe. (G) Djarkutan “Palace” section and ash pit location. (H) Molleli section and ash pit location.

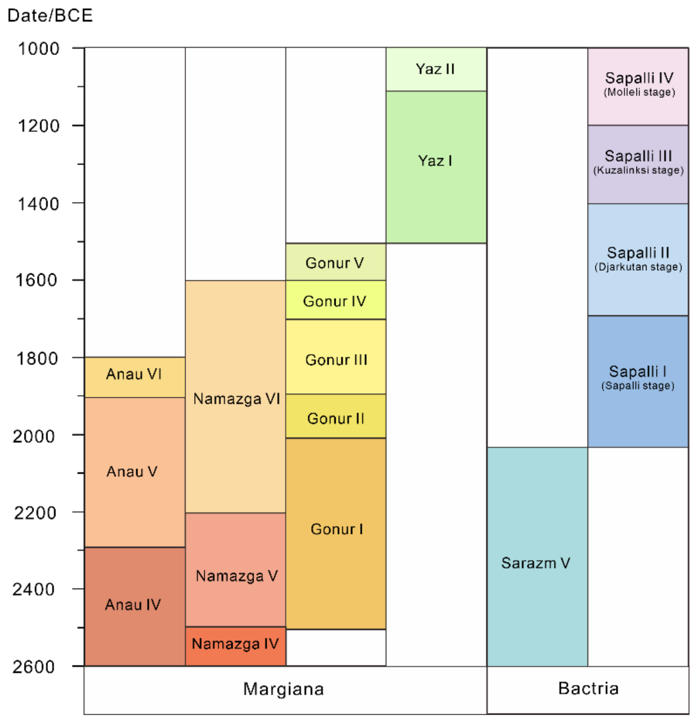

Fig. S3. The chronology sequence of different culture types within BMAC (*23*, *101–105*).

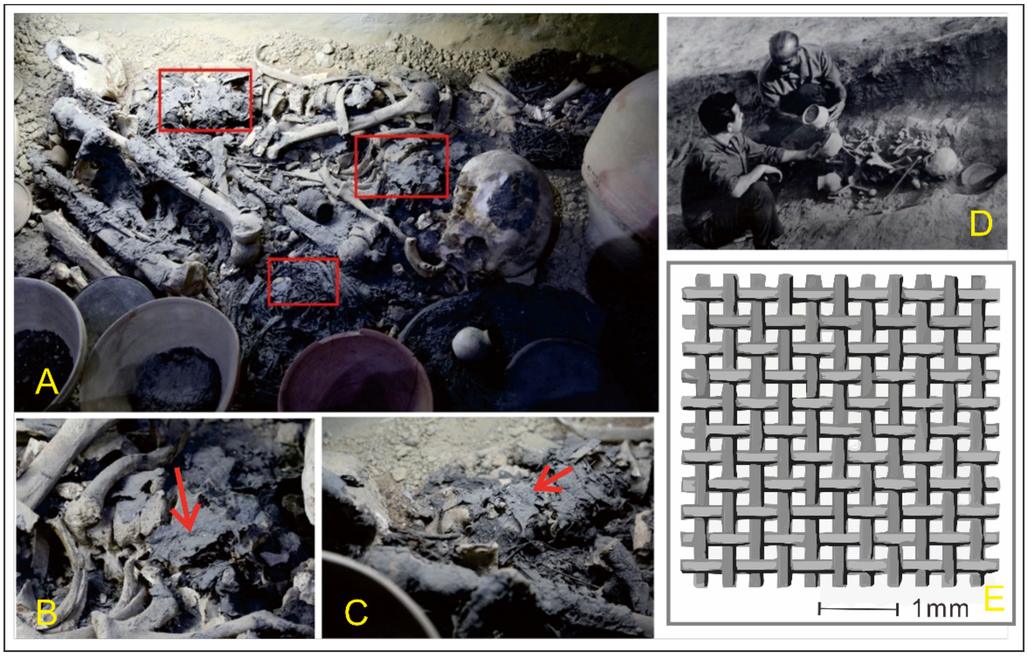

Fig. S4. Gravel No 1. (ca. 2000 BCE) from Sapalli Tepe, including clothing, very likely to be silk (marked with red boxes and arrows in **A**, **B**, and **C**), kept in the National Museum of Uzbekistan. (**D**) Excavation scene from 1969 showing archaeological materials from FAN (*24*), and (**E**) The model plan for a plain tabby silk fabric.

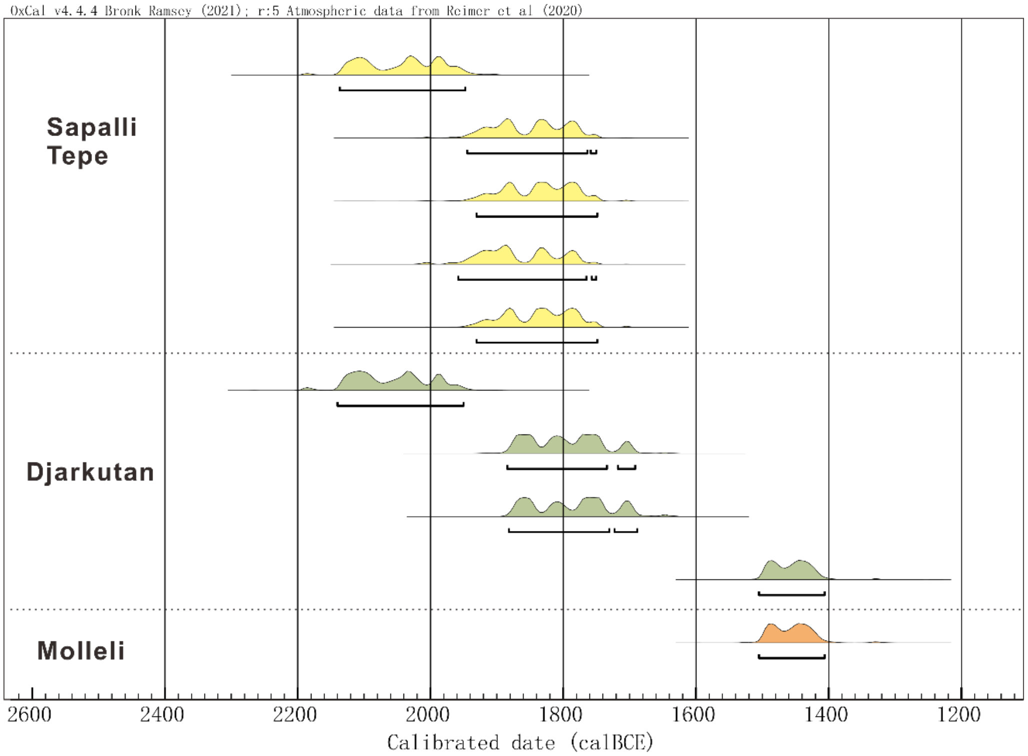

Fig. S5. The Chronology of Sapalli Tepe, Djarkutan and Molleli.

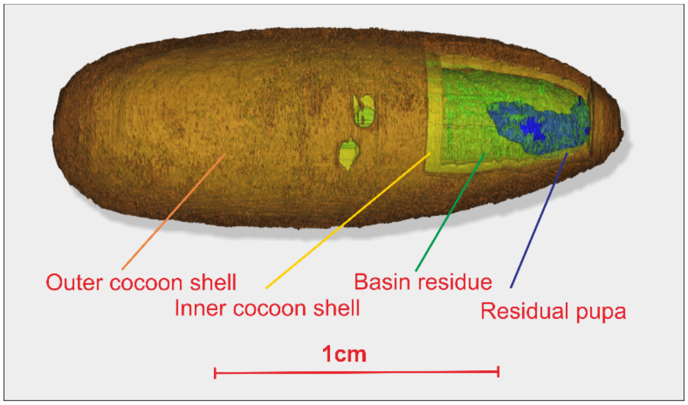

Fig. S6. Computed Tomography Scan reconstructed anatomical structure of the ancient cocoon (Sapalli-C-3).

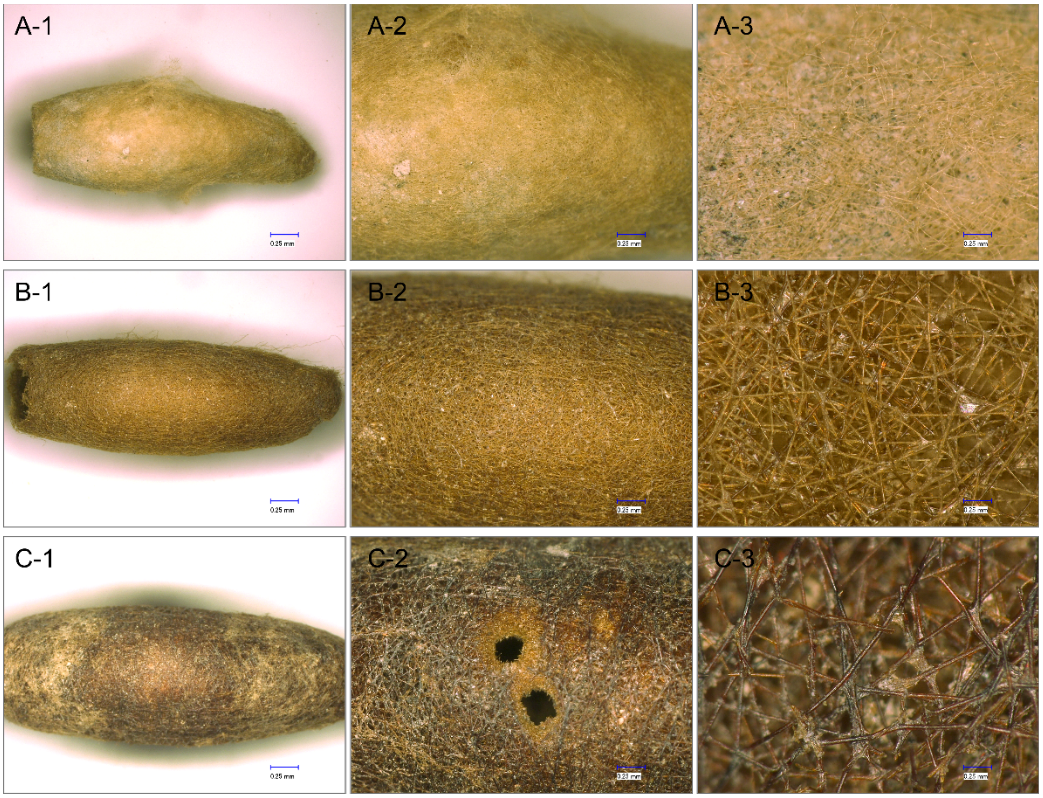

Fig. S7. The structure of the ancient silkworm cocoons under the microscope. (A-C) represent Sapalli-C-1 through 3, respectively. The scale bar is 0.25mm.

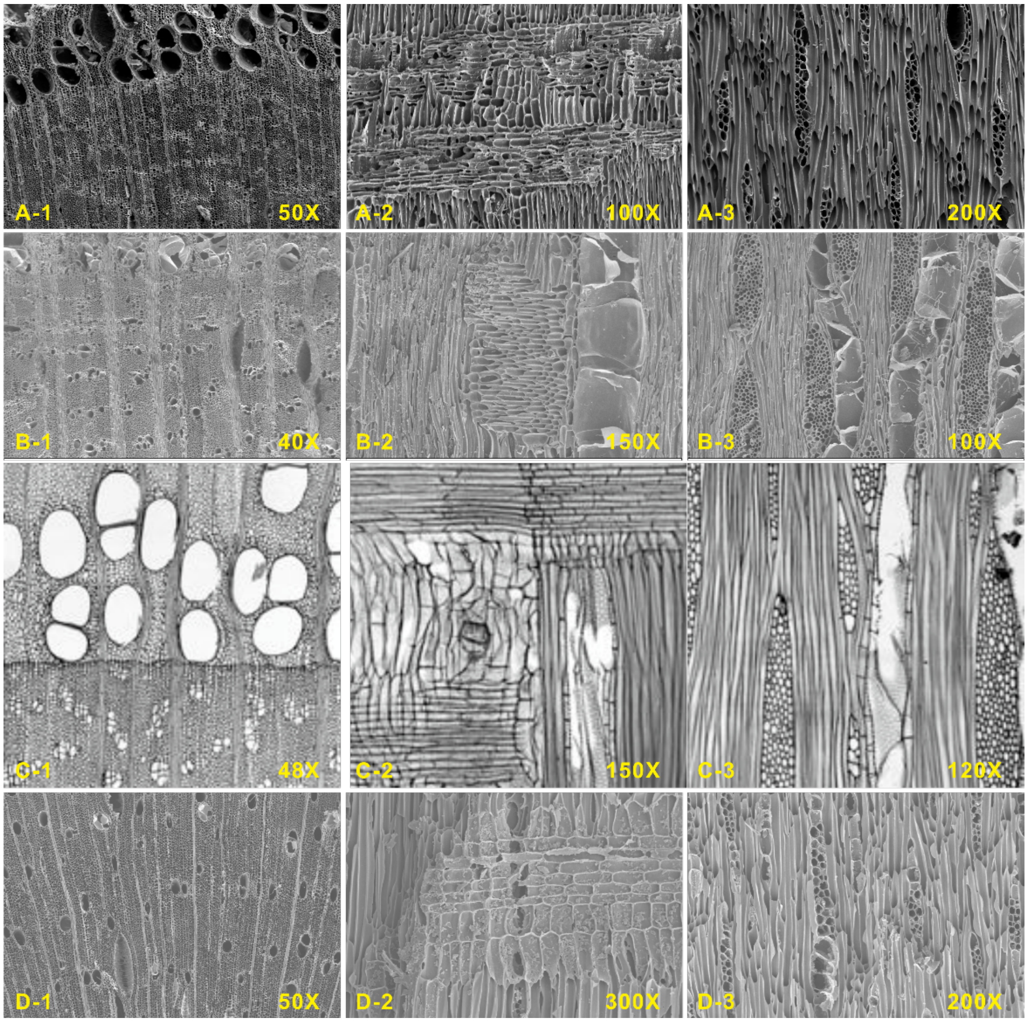

Fig. S8. The comparative wood anatomy of white and black mulberry under a scanning electron microscope. (**A**) *Morus* sp. wood charcoal found at Sapalli Tepe. (**B**) Modern white mulberry (*Morus alba*) collected in this study. (**C**) White mulberry wood anatomy (*57*). (**D**) Modern black mulberry (*Morus nigra*) collected in this study.

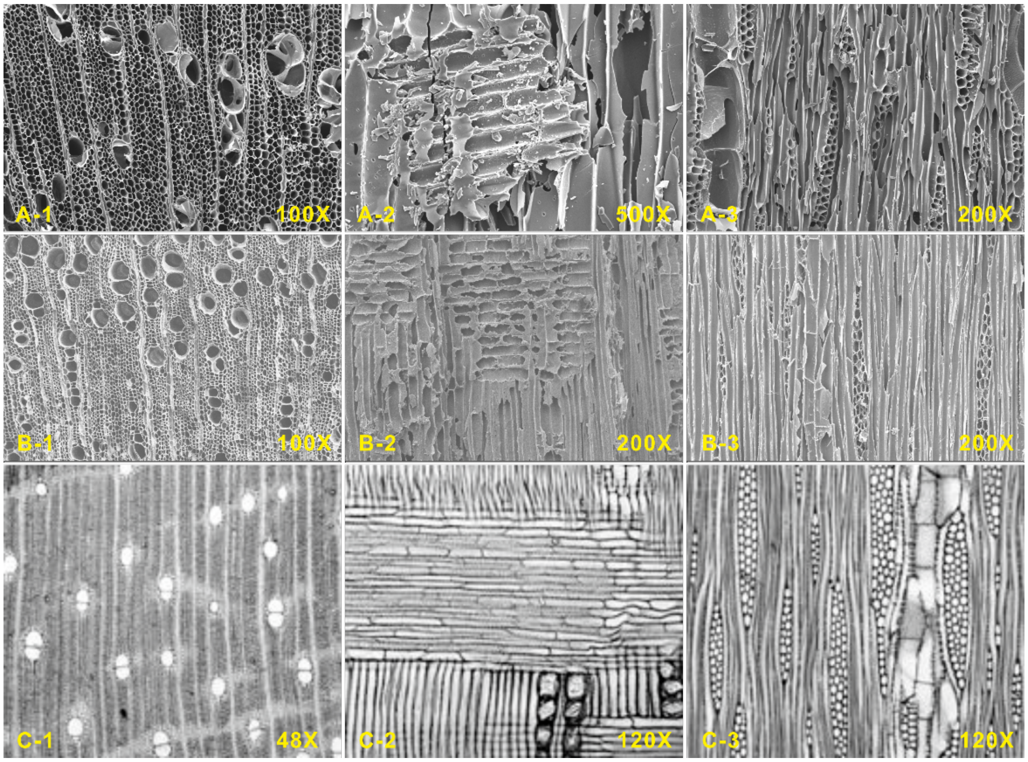

Fig. S9. The comparative wood anatomy of a *Citrus* plant under scanning electron microscope. (**A**) *Citrus* sp. wood charcoal found in the Sapalli Tepe. (**B**) Modern citron (*Citrus medica*) collected in this study. (**C)** Pummelo (*Citrus* × *aurantium*) wood anatomy (*57*).

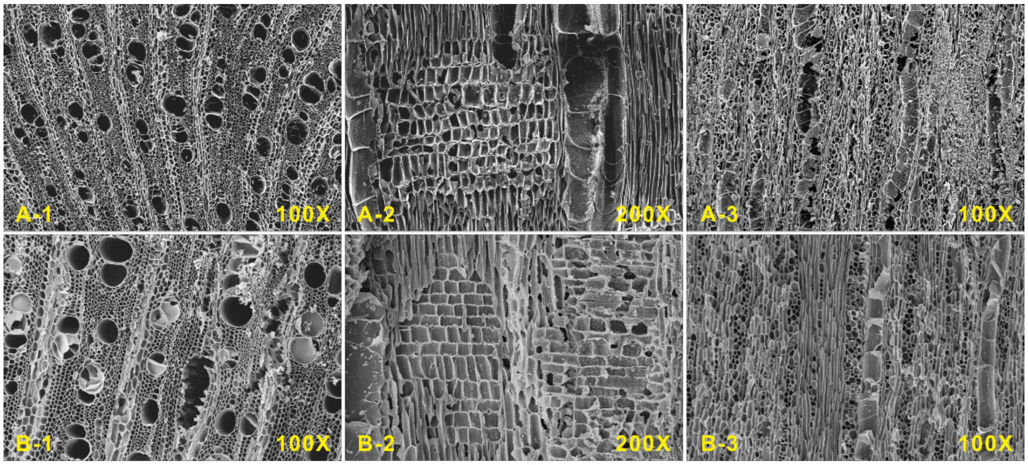

Fig. S10. The comparative wood anatomy of a fig tree under scanning electron microscope. (**A**) *Ficus* sp. wood charcoal found at Sapalli Tepe. (**B)** Modern fig (*Ficus carica*) collected in this study.

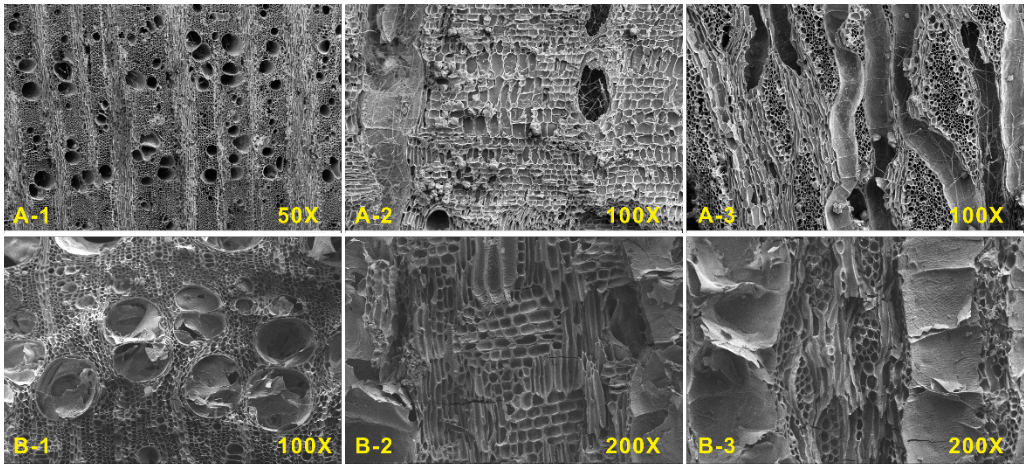

Fig. S11. The wood anatomy of *Tamarix* sp. (A) and broadleaf1 wood (B) under scanning electron microscope.

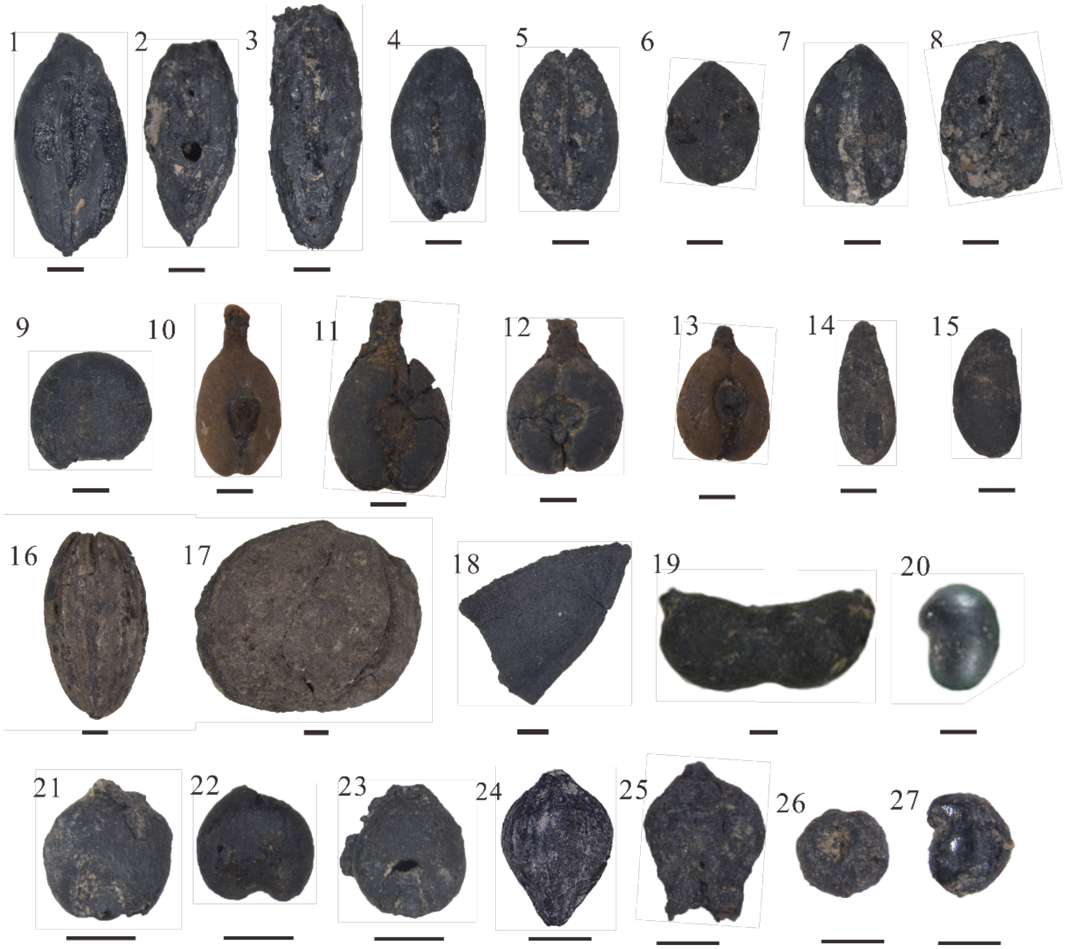

Fig. S12. Both charred and uncarbonized ancient seeds from Sapalli Tepe. 1-3 *Hordeum vulgare*; 4-7 *Hordeum vulgare* var. *nudum*; 8 *Triticum aestivum*; 9 *Vicia lens*; 10-13 *Vitis vinifera*; 14-15 *Linum usitatissimum*; 16 *Elaeagnus angustifolia*; 17 *Prunus* sp.; 18 *Pistacia vera*; 19-20 *Alhagi camelorum*; 21-23 *Panicum miliaceum*; 24 *Persicaria* sp.; 25 *Euclidium syriacum*; 26 *Galium spurium*; 27 Amaranthaceae; The scale bar is 1mm.

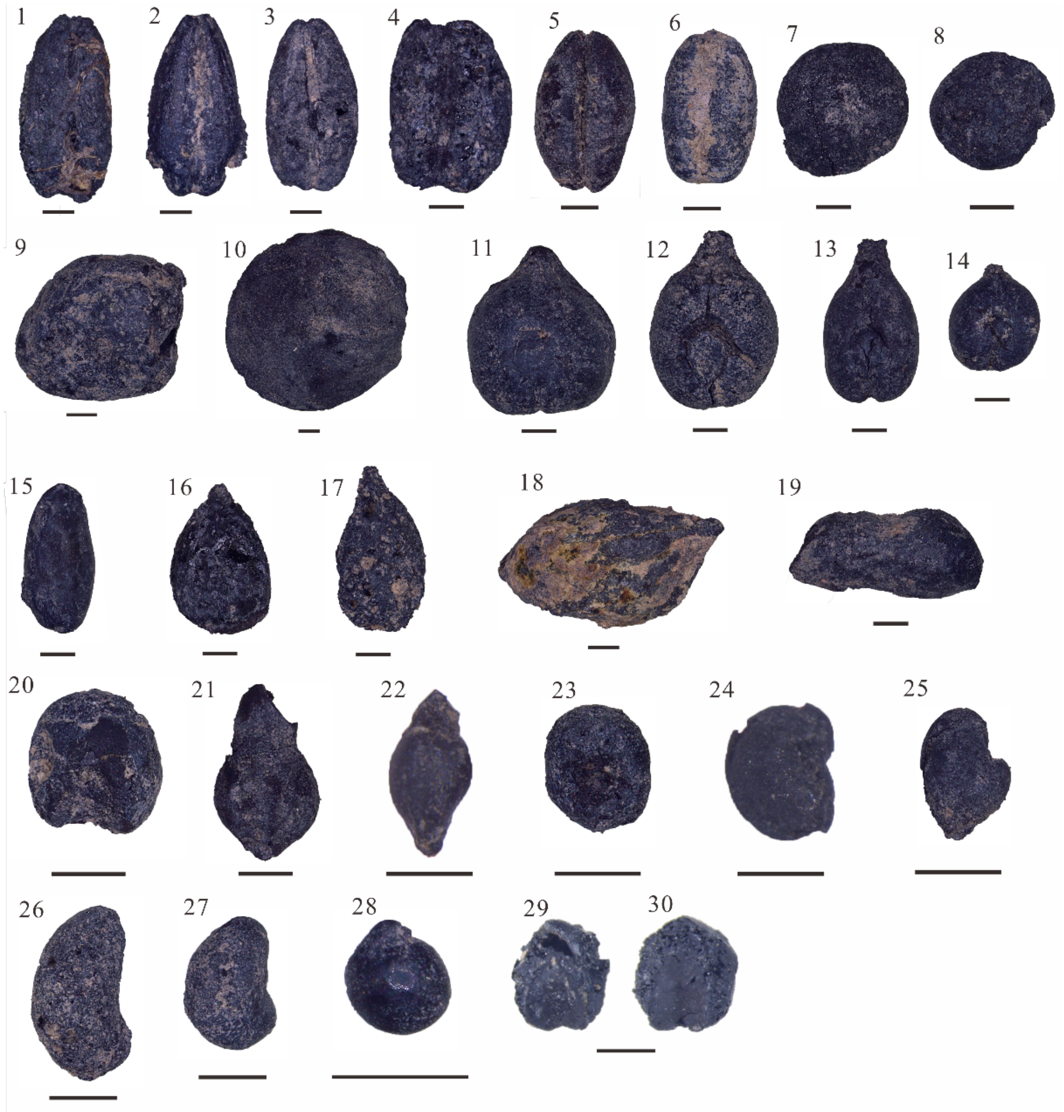

Fig. S13. Charred seeds from Djarkutan and foxtail millet from Molleli. 1-3 *Hordeum vulgare*; 4 *Triticum aestivum*; 5-6 *Hordeum vulgare* var. *nudum*; 7 *Pisum sativum*; 8 *Vicia lens*; 9 *Lathyrus sativus*; 10 *Prunus* sp.; 11-14 *Vitis vinifera*; 15 *Linum usitatissimum*; 16-17 *Malus* sp.; 18 *Elaeagnus angustifolia*; 19 *Alhagi camelorum*; 20 *Panicum miliaceum*; 21 *Euclidium syriacum*; 22 *Cyperus* sp.; 23 *Galium spurium*; 24-27 *Medicago* Type; 28 Amaranthaceae; 29-30 *Setaria italica*; The scale bar is 1mm.

Table S1. The pre-Han silk and silk-like remains found across the Old World.

Site	Date (BCE)	Method	Detected material or species
Qianshanyang	ca. 2750 or 2200–2000	morphological observation	Previous excavation reports the discovery of *Bombyx* silk remains(*63*, *64*); while no silk fiber tested from the newly discovered textile(*65*).
Jiahu	ca. 6500	mass spectrometry and proteomics analysis	silk fibroin (*2*).
Wanggou	ca. 4000	enzyme-linked immunosorbent assay	silk fibroin (*4*).
Qingtai	ca. 3500	morphological observation	*Bombyx* silk (*62*).
Xiyingcun	ca. 5000–3000	morphological observation	It was identified as *Bombyx mori* (*59*)and later analysis suggests that it is *Ronditia menaciana* (*60*, *61*).
Pazyryk	ca. 400	morphological observation	The yellow blouse was originally thought to be made of tussar silk; later research assigns it as a wild silk (*71*).
Quman	ca. 500	dye testing and weaving structure analysis	The fiber materials include *Bombyx* silk and wool (*70*).
Sapalli Tepe	ca. 1800	morphological observation	The fibers are probably silk (*24*).
Harrapan	2450–2000	morphological observation	The fibers are made from two different species of silkmoth (*Antheraea* sp.) (*11*).
Chanudaro	2600–2200	morphological observation	the silk is yet unidentified species, possibly an Eri silk (*Philosamia* spp.) (*11*).
Nevassa	1500–1050	morphological observation	A single thread was reported as silk (*72*).
Gordion	ca. 700	morphological observation	The darker threads appear to be silk (*14*).
Sardis	ca. 600–500	morphological observation	Greenewalt et al. report it as mohair (*74*); Good thinks it could be possibly silk due to the fineness of the fibers (*14*).
Kerameikos	ca. 500–400	morphological observation; protein and amino acid compositional analysis	Thorn's analysis suggests the samples come from five different textiles, namely silk of the *Bombyx mori* variety (*75*); proteomics study by Good shows it is wild silk from *Pachypasa otus* moth, native to the Mediterranean (*14*, *76*, *77*); Margariti et al. illustrate that they are cellulosic bast and some of them are possible cotton fibers (*16*).
Akrotiri	ca. 1600	morphological observation	cocoon of *Pachypasa otus* (*73*)

Deir al Medina	ca. 1000	morphological observation	It was regarded as wild silk with strong possibility by Good (*14*), while it is questioned by later examination by Good (*13*).
Hohmichele	ca. 500	amino acid analyses and morphological observation	It was identified as vegetable fibers in Riek and Hundt (*78*); later identification refuted that it is silk by Banck-Burgess (*79–81*) and Mann (*82*); Good suggested that it is silk (*14*, *77*).
Hochdorf	ca. 530	amino acid analyses	Hundt mentioned that the fibers are similar to that from Hohmichele grave VI which had been identified as silk (*83*); while proteomics by Mann found no evidence of silk (*82*).
Altrie	450-25	morphological observation	Good set it as silk (*14*)while all the previous studies by Heyart and Thill classified it as wool (*84*, *85*).

Table S2. The characteristics and comparison of different culture types within BMAC

Region	Culture	Cultural characteristics
Margiana	Anau	This period represents a transitional phase between the Neolithic and Chalcolithic. Settlements remained relatively small in scale. The material culture is characterized by handmade pottery, with the potter's wheel in later phases. The painted motifs were predominantly composed of rhomboid and triangular designs (*106*)
	Namazga	This culture succeeded the Anau. Pottery was predominantly wheel-made, with painted decoration becoming simplified and later largely replaced by plain, polished ware. Red copper was utilized during the early period. Settlements exhibited evidence of functional zoning, and by Phase IV, incipient urban centers had emerged(*107*).
	Gonur	The culture exhibited a high level of urbanization, characterized by palaces, temples, reservoirs, and royal necropolis. Wheel-made, plain and polished pottery constituted the predominant ceramic tradition. A highly developed bronze industry produced tools, weapons, and ritual objects(*20*).
	Yaz	The settlements of this culture are organized around defensive fortifications and irrigation systems. The pottery is characterized by handmade vessels decorated with geometric patterns. The metallurgy was marked by the concurrent use of bronze and iron(*108*).
Bactria	Sarazm	Settlements were relatively large in scale, featuring functional zoning and irrigation systems. The pottery is characterized by handmade red ware, with wheel-thrown vessels appearing during later phases. Bronze artifacts were primarily utilitarian tools(*109*).
	Sapalli	Proto-urban centers emerged, characterized by complex settlement structures and internal functional differentiation. The ceramic assemblage is characterized by wheel-made grey and red pottery, decorated with impressed and painted motifs. Bronze technology was highly advanced, supporting the production of sophisticated tools, weapons, and ritual implements(*22*).

Table S3. radiocarbon age data and the dating marital of the three sites (SPL=Sapalli Tepe, JLGD= Djarkutan).

Lab No	Sample No	Marital	Measured age	Calibrated Age (2δ, BC)
459967	SPL-4	millet seed	3520 +/- 30 BP	1930–1750
459966	SPL-3-1	cocoon	3530 +/- 30 BP	1940–1765
459965	SPL-3	millet seed	3520 +/- 30 BP	1930–1750
459964	SPL-1	wheat seed	3540 +/- 30 BP	1945–1865 and 1850–1770
400298	SPL-2	charcoal	3660 +/- 30 BP	2135–1945
459963	JLGD-BA-5	wheat seed	3470 +/- 30 BP	1885–1730 and 1715–1690
459962	JLGD-BA-1	wheat seed	3460 +/- 30 BP	1880–1690
459961	JLGD-MBA-1	millet seed	3180 +/- 30 BP	1505–1410
459960	JLGD-EBA-2	wheat seed	3670 +/- 30 BP	2140–1955
462797	Molleli-01	wheat seed	3180 +/- 30 BP	1507–1407

Table S4. Proteins identified by LC-MS/MS

Sample	Identified protein	Species	Unique peptides	Sequence coverage (%)	iBAQ	SilkDB ID	NCBI ID
Sapalli-C-1	30k protein Bmlp2	*Bombyx mori*	6	35.6	42632000	BGIBMGA004399	NP_001095198
	30k protein Bmlp1	*Bombyx mori*	3	17.2	12462000	BGIBMGA004394	NP_001037486
	30k protein Bmlp6	*Bombyx mori*	4	16.2	11860000	BGIBMGA004457	NP_001095196
	sex-specific storage protein SP2	*Bombyx mori*	3	4.0	2605500	BGIBMGA009028	NP_001037590
	fibroin heavy chain	*Bombyx mori*	2	0.6	1900100	BGIBMGA005111	NP_001106733
	sericin 1	*Bombyx mori*	5	6.9	990330	BGIBMGA001793	NP_001037506
	sex-specific storage protein SP1	*Bombyx mori*	2	3.7	65884	BGIBMGA011266	NP_001106747
Sapalli-C-2	30k protein Bmlp2	*Bombyx mori*	4	19.7	132400000	BGIBMGA004399	NP_001095198
	30k protein Bmlp6	*Bombyx mori*	4	16.2	56799000	BGIBMGA004457	NP_001095196
	30k protein Bmlp1	*Bombyx mori*	3	17.2	10240000	BGIBMGA004394	NP_001037486
	sex-specific storage protein SP2	*Bombyx mori*	3	5.7	2892800	BGIBMGA009028	NP_001037590
	sericin 1	*Bombyx mori*	2	3.0	486440	BGIBMGA001793	NP_001037506
Sapalli-C-3	30k protein Bmlp2	*Bombyx mori*	5	28.0	37413000	BGIBMGA004399	NP_001095198
	30k protein Bmlp1	*Bombyx mori*	3	17.2	9013500	BGIBMGA004394	NP_001037486
	30k protein Bmlp6	*Bombyx mori*	3	15.8	7630600	BGIBMGA004457	NP_001095196
	sex-specific storage protein SP2	*Bombyx mori*	4	7.1	6058300	BGIBMGA009028	NP_001037590
	sex-specific storage protein SP1	*Bombyx mori*	3	6.2	255810	BGIBMGA011266	NP_001106747

REFERENCES

1. Q. Xia, Y. Guo, Z. Zhang, D. Li, Z. Xuan, Z. Li, F. Dai, Y. Li, D. Cheng, R. Li, T. Cheng, T. Jiang, C. Becquet, X. Xu, C. Liu, X. Zha, W. Fan, Y. Lin, Y. Shen, L. Jiang, J. Jensen, I. Hellmann, S. Tang, P. Zhao, H. Xu, C. Yu, G. Zhang, J. Li, J. Cao, S. Liu, N. He, Y. Zhou, H. Liu, J. Zhao, C. Ye, Z. Du, G. Pan, A. Zhao, H. Shao, W. Zeng, P. Wu, C. Li, M. Pan, J. Li, X. Yin, D. Li, J. Wang, H. Zheng, W. Wang, X. Zhang, S. Li, H. Yang, C. Lu, R. Nielsen, Z. Zhou, J. Wang, Z. Xiang, J. Wang, Complete resequencing of 40 genomes reveals domestication events and genes in silkworm (*Bombyx*). *Science* **326**, 433–436 (2009).

2. Y. Gong, L. Li, D. Gong, H. Yin, J. Zhang, Biomolecular evidence of silk from 8,500 years ago. *PLOS ONE* **11**, e0168042 (2016).

3. H. Xiang, X. Liu, M. Li, Y. Zhu, L. Wang, Y. Cui, L. Liu, G. Fang, H. Qian, A. Xu, W. Wang, S. Zhan, The evolutionary road from wild moth to domestic silkworm. *Nat. Ecol. Evol.* **2**, 1268–1279 (2018).

4. F. Zhao, Ed., *Silk Journey: Chinese Silk and the Silk Road* (Huangshan Publishing House, Hefei, 2021).

5. C.-X. Yang, S.-Y. Liu, N. J. C. Zerega, G. W. Stul, E. M. Gardner, Q. Tian, W. Gu, Q. Lu, R. A. Folk, H. R. Kates, R. P. Guralnick, D. E. Solti, P. S. Soltis, Y.-H. Wang, T.-S. Yi, Phylogeny and biogeography of *Morus* (Moraceae). *Agronomy* **13**, 2021 (2023).

6. X. Liu, P. J. Jones, G. M. Matuzeviciute, H. V. Hunt, D. L. Lister, T. An, N. Przelomska, C. J. Kneale, Z. Zhao, M. K. Jones, From ecological opportunism to multi-cropping. *Quat. Sci. Rev.* **206**, 21–28 (2019).

7. R. N. Spengler, Ed. *Fruit from the Sands: The Silk Road Origins of the Foods We Eat* (Univ. California Press, Oakland, CA, 2019).

8. X. Zhou, J. Yu, R. N. Spengler, H. Shen, K. Zhao, J. Ge, Y. Bao, J. Liu, Q. Yang, G. Chen, P. W. Jia, X. Li, 5,200-year-old cereal grains from the eastern Altai Mountains redate the trans-Eurasian crop exchange. *Nat. Plants* **6**, 78–87 (2020).

9. I. Good, Archaeological textiles: A review of current research. *Ann. Rev. Anthropol.* **30**, 209–226 (2001).

10. R. S. Peigler, "Wild silks: Their entomological aspects and their textile applications" in *Handbook of Natural Fibres (Second Edition)*, R. M. Kozłowski, M. Mackiewicz-Talarczyk, Eds. (Woodhead Publishing, Cambridge, 2020), pp. 715–745.

11. I. Good, J. M. Kenoyer, R. H. Meadow, New evidence for early silk in the Indus Civilization. *Nat. Prec.* **2008**, 10.1038/npre.2008.1900.1 (2008).

12. G. M. Richter, Silk in Greece. *Am. J. Archaeol.* **33**, 27–33 (1929).

13. I. Good, "The archaeology of early silk" in *Textile Society of America Symposium Proceedings* (Textile Society of America, Millersville, MD, 2002).

14. I. Good, On the question of silk in pre-Han Eurasia. *Antiquity* **69**, 959–968 (1995).

15. L. Bender Jørgensen, The question of prehistoric silks in Europe. *Antiquity* **87**, 581–588 (2013).

16. C. Margariti, S. Protopapas, V. Orphanou, Recent analyses of the excavated textile find from Grave 35 HTR73, Kerameikos cemetery, Athens. *Greece. J. Archaeol. Sci.* **38**, 522–527 (2011).

17. N. K. Adams, W. S. Webb, "Silk in Ancient Nubia: One Road, Many Sources" in *Silk Roads, Other Roads: Textile Society of America 8th Biennial Symposium* (Smith College, Northampton, MA, 2002), pp. 81–91.

18. B. Hildebrandt, The terminology of silks in texts of the Roman Empire: Qualities, origins, products, and uses. *Acta Via Ser.* **6**, 117–139 (2021).

19. R. S. Peigler, Wild silks of the world. *Am. Entomol.* **39**, 151–162 (1993).

20. V. I. Sarianidi, G. Puschnigg, The fortification and palace of northern Gonur. *Iran* **40**, 75–87 (2002).

21. H. P. Francfort, “The early periods of Shortughai (Harappan) and the western Bactrian culture of Dashly” in *South Asian Archaeology 1981* (Cambridge Univ. Press, Cambridge, 1984), pp. 170–175.

22. A. A. Askarov, Southern Uzbekistan in the second millennium D.C. *Sov. Anthropol. Archaeol.* **19**, 256–272 (1981).

23. A. H. Dani, V. M. Masson, Eds., *History of Civilizations of Central Asia. Volume I: The Dawn of Civilization: Earliest Times to 700 B.C.* (UNESCO Publishing, Paris, 1992).

24. A. A. Askarov, Ed., *Sapalli Tepe* (FAN, Tashkent, 1973).

25. A. A. Askarov, Ed., *Drevenezemledel’cheskaja Kul’tura Bronzovogo Veka Juzhnogo Uzbekistana* [The Ancient Bronze Age Farmers’ Culture of Southern Uzbekistan] (FAN, Tashkent, 1977).

26. L. P’yankova, Central Asia in the Bronze Age. *Antiquity* **68**, 355–372 (1994).

27. A. Razzokov, Ed., *Sarazm* (Echod, Dushanbe, 2008).

28. A. A. Askarov, B. Abdullaev, Eds., *Djarkutan (K Probleme Protogorodskoj Tsivilizatsii Na Juge Uzbekistana)* [Djarkutan (On the Question of a Proto-Urban Civilization in Southern Uzbekistan)] (FAN, Tashkent, 1983).

29. A. A. Askarov, T. Shirinov, The “palace,” temple, and necropolis of Jarkutan. *Bull. Asia Inst.* **8**, 13–25 (1994).

30. E. Karami, K. Pourtahmasi, S. Shahverdi, Wood anatomical structure of *Morus alba* L. and *Morus nigra* L., native to Iran. *Not. Sci. Biol.* **2**, 129–132 (2010).

31. S. Muthukumaran, Ed., *The Tropical Turn: Agricultural Innovation in the Ancient Middle East and the Mediterranean* (Univ. California Press, Oakland, CA, 2023).

32. Z. H. Xiang, J. T. Huang, J. G. Xia, C. Lu, Eds., *Biology of Sericulture* (China Forestry Publishing House, Beijing, 2005).

33. H. Zheng, R. Yang, J. Guo, Z. Xie, J. Liu, H. Yang, L. Jia, J. Yu, Q. Cai, F. Tang, Y. Zhou, Evidence of the use of silk by bronze age civilization for sacrificial purposes in the Yangtze River basin of China. *Sci. Rep.* **14**, 29175 (2024).

34. Z.-Y. Yu, The excavation report of cemetery M8(95MNI) at Niya site in Minfeng County. *Xinjiang. Cult. Relics* **1**, 4–40 (2002).

35. J. E. Hill, Ed. *Through the Jade Gate to Rome: A Study of the Silk Routes during the Later Han Dynasty 1st to 2nd Centuries CE* (BookSurge Publishing, Charleston, SC, 2009), pp. 466–467.

36. V. I. Sarianidi, Temples of Bronze Age Margiana. *Antiquity* **68**, 388–397 (1994).

37. R. N. Spengler, Agriculture in the Central Asian Bronze Age. *J. World Prehist.* **28**, 215–253 (2015).

38. B. Mir-Makhamad, S. Stark, S. Mirzaakhmedov, H. Rahmonov, R. N. Spengler, Food globalization in southern Central Asia: Archaeobotany at Bukhara between antiquity and the Middle Ages. *Archaeol. Anthropol. Sci.* **15**, 124 (2023).

39. A. Tikader, Distribution, diversity, utilization and conservation of mulberry (*Morus* spp.) in North West of India. *Asian Australas. J. Plant Sci. Biotechnol.* **5**, 67–72 (2011).

40. A. Livarda, M. van der Veen, Social access and dispersal of condiments in North-West Europe from the Roman to the medieval period. *Veg. Hist. Archaeobotany* **17**, 201–209 (2008).

41. J. M. Marston, L. Castellano, Crop introductions and agricultural change in Anatolia during the long first millennium CE. *Veg. Hist. Archaeobotany* **35**, 129–142 (2026).

42. S. Saraswat, “Banawali (29°37′5″; 75°23′6″), District Hissar” in *Indian Archaeology 1996–1997: A Review* (Archaeological Survey of India, New Delhi, 2002), pp. 203.

43. E. Asouti, D. Q. Fuller, Eds., *Trees and Woodlands of South India: Archaeological Perspectives* (Munshiram Manoharlal Publishers, New Delhi, 2008), p. 126.

44. F. A. Lone, M. Khan, G. M. Buth, Eds., *Palaeoethnobotany: Plants and Ancient Man in Kashmir* (CRC Press, Boca Raton, FL, 1993).

45. H. Shen, X. Zhou, A. Betts, P. W. Jia, K. Zhao, X. Li, Fruit collection and early evidence for horticulture in the Hexi Corridor, NW China, based on charcoal evidence. *Veg. Hist. Archaeobotany* **28**, 187–197 (2019).

46. H. E. Jiang, Ed., *Agricultural Activities and Plant Utilization of the Ancient Yanghai People, Turpan of Xinjiang, China* (Science Press, Beijing, 2022).

47. H. Shen, R. N. Spengler, X. Zhou, A. Betts, P. W. Jia, K. Zhao, X. Li, Seeing the wood for the trees: Active human–environmental interactions in arid northwestern China. *Earth Syst. Sci. Data* **16**, 2483–2499 (2024).

48. G. A. Wu, J. Terol, V. Ibanez, A. López-García, E. Pérez-Román, C. Borredá, C. Domingo, F. R. Tadeo, J. Carbonell-Caballero, R. Alonso, F. Curk, D. Du, P. Ollitrault, M. L. Roose, J. Dopazo, F. G. Gmitter, D. S. Rokhsar, M. Talon, Genomics of the origin and evolution of *Citrus*. *Nature* **554**, 311–316 (2018).

49. Y. Huang, J. He, Y. Xu, W. Zheng, S. Wang, P. Chen, B. Zeng, S. Yang, X. Jiang, Z. Liu, L. Wang, X. Wang, S. Liu, Z. Lu, Z. Liu, H. Yu, J. Yue, J. Gao, X. Zhou, C. Long, X. Zeng, Y.-J. Guo, W.-F. Zhang, Z. Xie, C. Li, Z. Ma, W. Jiao, F. Zhang, R. M. Larkin, R. R. Krueger, M. W. Smith, R. Ming, X. Deng, Q. Xu, Pangenome analysis provides insight into the evolution of the orange subfamily and a key gene for citric acid accumulation in citrus fruits. *Nat. Genet.* **55**, 1964–1975 (2023).

50. C. J. Stevens, C. Murphy, R. Roberts, L. Lucas, F. Silva, D. Q. Fuller, Between China and South Asia: A Middle Asian corridor. *Holocene* **26**, 1541–1555 (2016).

51. D. Q. Fuller, C. J. Stevens, Between domestication and civilization: The role of agriculture and arboriculture in the emergence of the first urban societies. *Veg. Hist. Archaeobotany* **28**, 263–282 (2019).

52. X. Liu, D. L. Lister, Z. Zhao, C. A. Petrie, X. Zeng, P. J. Jones, R. A. Staff, A. K. Pokharia, J. Bates, R. N. Singh, S. A. Weber, G. M. Matuzeviciute, G. Dong, H. Li, H. Lü, H. Jiang, J. Wang, J. Ma, D. Tian, G. Jin, L. Zhou, X. Wu, M. K. Jones, Journey to the east: Diverse routes and variable flowering times for wheat and barley en route to prehistoric China. *PLOS ONE* **12**, e0187405 (2017).

53. B. Cerasetti, L. Rouse, R. Arciero, T. Billings, M. Carra, A. Curci, J. de Grossi Mazzorin, L. Forni, É. Luneau, A. Potenza, R. N. Spengler, "The rise and decline of the desert cities: The last stages of the BMAC at Togolok 1" in *Cultures in Contact*, C. Baumer, M. Novák, S. Rutishauser, Eds. (Harrassowitz Verlag, Wiesbaden, 2022), pp. 89–116.

54. S. Salvatori, M. Tosi, Eds., *The Bronze Age and Early Iron Age in the Margian Lowlands: Facts and Methodological Proposals for a Redefinition of the Research Strategies. Archaeological Map of the Murghab Delta Studies and Reports, vol. II. BAR International Series 1806* (Archaeopress, Oxford, 2008).

55. B. Lyonnet, N. A. Dubova, "Questioning the Oxus Civilization or Bactria-Margiana Archaeological Culture (BMAC): An overview" in *The World of the Oxus Civilization* (Routledge, London, 2020), pp. 7–65.

56. Y. Zhang, P. Zhao, Z. Dong, D. Wang, P. Guo, X. Guo, Q. Song, W. Zhang, Q. Xia, Comparative proteome analysis of multi-layer cocoon of the silkworm *Bombyx mori*. *PLOS ONE* **10**, e0123403 (2015).

57. A. Fahn, E. Werker, P. Baas, Eds., *Wood Anatomy and Identification of Trees and Shrubs from Israel and Adjacent Regions* (Israel Academy of Sciences and Humanities, Jerusalem, 1986).

58. J. Q. Cheng, J. J. Yang, P. Liu, Eds., *Atlas of Wood in China* (China Forestry Publishing House, Beijing, 1992).

59. J. Li, Ed., *The Prehistoric Remains of His-yin Ts'un, A Preliminary Report* (Tsinghua University Research Institute, Beijing, 1927).

60. D. Kuhn, “The silk workshops of the Shang Dynasty (16th–11th Century BC)” in *Explorations in the History of Science and Technology in China*, D. Hu, Ed. (Shanghai Classics Publishing House, Shanghai, 1982), pp. 367–408.

61. E. J. W. Barber, Ed., *Prehistoric Textiles* (Princeton Univ. Press, Princeton, NJ, 1991).

62. H. L. Zhang, H. Y. Gao, Observation and analysis on the silk materials from Qingtai site, Xingyang. *Cult. Relics Cent. China* **3**, 10–16 (1999).

63. K. M. Zhou, The revelation on the silk remains from the Qianshanyang site. *Cult. Relics* **1**, 74–77 (1980).

64. H. Xu, Q. M. Ou, M. S. Li, H. Z. Zhang, The test on the silk materials from Qianshanyang site. *Silk Mon.* **2**, 43–45 (1981).

65. Y. Zhou, The origin of silk—The Qianshanyang site from Huzhou. *Silk Mon.* **6**, 1–5 (2006).

66. Zhejiang Provincial Institute of Cultural Relics and Archaeology, Ningbo Municipal Institute of Cultural Heritage Management, The Hemudu Site Museum of Yuyao City, *Excavation Report of the Neolithic Site—Hemudu* (Cultural Relics Press, Beijing, 2003).

67. The Shanxi Archaeological Team, Institute of Archaeology, Academic Sinica, Excavations of the Neolithic sites at Dongzhuang Cun and Xiwang Cun in Ruicheng County, Shanxi Province. *Acta Archaeol. Sin.* **1**, 1–63 (1973).

68. School of Archaeology Jilin University, Shanxi Provincial Academy of Archaeology, Yuncheng Cultural Relics Conservation Center, Excavation during 2019–2020 of the Neolithic site at Shicun in Xiaxian, Shanxi. *World Antiq.* **2**, 2–7 (2021).

69. K. Riboud, “A closer view of early Chinese silks” in *Studies in Early Textiles*, M. Gervers, Ed. (Univ. Toronto Press, Toronto, 1977), pp. 89–98.

70. Y. Zhou, L. L. Jia, J. Liu, Scientific research on the textiles excavated from a Zoroastrian cemetery in Pamir. *Sci. Conserv. Archaeol.* **31**, 55–64 (2019).

71. P. G. Bahn, Ed., *The Atlas of World Geology* (Checkmark Books, New York, 2000), p. 128.

72. A. N. Gulati, “A note on the early history of silk in India” in *Technical Reports on Archaeological Remains III*, J. Clutton-Brock, Vishnu-Mittre, A. N. Gulati, Eds. (Deccan College, Pune, 1961), pp. 53–59.

73. E. Panagiotakopulu, P. C. Buckland, P. M. Day, C. Doumas, A. Sarpaki, P. Skidmore, A lepidopterous cocoon from Thera and evidence for silk in the Aegean Bronze Age. *Antiquity* **71**, 420–429 (1997).

74. C. H. Greenewalt, N. Cahill, H. Dedeoğlu, P. Herrmann, W. Rast, “The Sardis Campaign of 1986” in *Preliminary Reports of ASOR-Sponsored Excavations* (The John Hopkins University Press, 1990), pp. 137–177.

75. H.-J. Hundt, Über vorgeschichtliche Seidenfunde. *Jahrb. Römisch-Ger. Zent. Mus.* **16**, 59–71 (1970).

76. I. Good, “When east met west: Interpretative problems in assessing Eurasian contact and exchange in antiquity” in *New Directions in Silk Road Archaeology. Proceedings of a Workshop*, A. V. G. Betts, F. Kidd, Eds. (Dietrich Reimer, Berlin, 2010), pp. 35–48.

77. I. Good, “Strands of connectivity: Assessing the evidence for long distance exchange of silk in Later Prehistoric Eurasia” in *Interweaving Worlds: Systemic Interactions in Eurasia, 7th–1st Millennia BC*, T. C. Wilkinson, S. Sherratt, J. Bennet, Eds. (Oxbow, Oxford, 2011), pp. 218–230.

78. G. Riek, H.-J. Hundt, Eds., *Der Hohmichele: Ein Fürstengrabhügel der späten Hallstattzeit bei der Heuneburg* (Römisch-Germanische Forschungen 25, de Gruyter, Berlin, 1962).

79. J. Banck-Burgess, “Die Textilfunde aus dem hallstattzeitlichen Fürstengrab von Hochdorf, Gemeinde Eberdingen (Kreis Ludwigsburg)” in *NESAT V. Textilsymposium Neumünster: Archäologische Textilfunde, 4–7.5.1993*, G. Jaacks, K. Tidow, Eds. (Textilmuseum Neumünster, Neumünster, 1994), pp. 43–52.

80. J. Banck-Burgess, Ed., *Hochdorf IV, Die Textilfunde aus dem späthallstattzeitlichen Fürstengrab von Eberdingen-Hochdorf (Kreis Ludwigsburg) und weitere Grabtextilien aus hallstatt-latènezeitlichen Kulturgruppen* (Forschungen und Berichte zur Vor- und Frühgeschichte 70, Konrad Theiss, Stuttgart, 1999).

81. J. Banck-Burgess, "Case study: The textiles from the princely burial at Eberdingen-Hochdorf, Germany" in *Textiles and Textile Production in Europe: From Prehistory to AD 400*, M. Gleba, U. Mannering, Eds. (Oxbow, Oxford, 2012), pp. 139–150.

82. K. Mann, "Aminosäurenanalysen" in *Hochdorf IV, Die Textilfunde aus dem späthallstattzeitlichen Fürstengrab von Eberdingen-Hochdorf (Kreis Ludwigsburg) und weitere Grabtextilien aus hallstatt-latènezeitlichen Kulturgruppen*, J. Banck-Burgess, Ed. (Forschungen und Berichte zur Vor- und Frühgeschichte 70, Konrad Theiss, Stuttgart, 1999), pp. 235–238.

83. H.-J. Hundt, "Die Textilien im Grab von Hochdorf" in *Der Keltenfürst von Hochdorf. Methoden und Ergebnisse der Landesarchäologie, Katalog zur Ausstellung Stuttgart, Kunstgebäude vom 14 August bis 13 Oktober 1985*, D. Planck, J. Biel, G. Süsskind, A. Wais, Eds. (Landesdenkmalamt Baden-Württemberg, Stuttgart, 1985), pp. 106–115.

84. H. Heyart, Textilreste. *Hémecht* **24**, 500–501 (1972).

85. G. Thill, "Altrier" in *Trésors des princes celtes*, J.-P. Mohen, A. Duval, C. Eluère, Eds. (Réunion des musées nationaux, Paris, 1987), pp. 251–254.

86. B. Cerasetti, R. Arciero, M. Carra, A. Curci, J. de Grossi Mazzorin, L. Forni, É. Luneau, L. M. Rouse, R. N. Spengler, "Bronze and Iron Age urbanization in Turkmenistan: Preliminary results from the excavation of Togolok 1" in *Urban Cultures of Central Asia from the Bronze Age to the Karakhanids*, C. Baumer, M. Novák, Eds. (Harrassowitz Verlag, Wiesbaden, 2019), pp. 63–72.

87. E. Luneau, Ed., *La Fin de la Civilisation de l'Oxus* (de Boccard, Paris, 2014).

88. S. Salvatori, "Thinking around Grave 3245 in the 'royal graveyard' of Gonur" in *Na Puti Otkrytija Tsivilizatsii*, P. M. Kozhin, M. F. Kosarev, N. A. Dubova, Eds. (Aletejja, St. Petersburg, 2010), vol. 3, pp. 244–257.

89. V. I. Sarianidi, N. A. Dubova, T. J. Vallée-Raevsky, Nouvelles sépultures sur le territoire de la "nécropole royale" de Gonur Dépé. *Arts Asiat.* **65**, 5–26 (2010).

90. N. A. Dubova, "The 'royal necropolis' at Gonur Depe" in *The World of the Oxus Civilization* (Routledge, London, 2020), pp. 334–364.

91. V. I. Sarianidi, "Drevnjaja Baktrija: Novye aspekty staroj problem" in *Etnicheskie Problemy Istorii Tsentral'noj Azii v Drevnosti* (Nauka, Moscow, 1981), pp. 180–191.

92. V. I. Sarianidi, "Ob odnoj gruppe drevnebaktrijskoj gliptiki" in *Drevnjaja Indija i Nauka* (Nauka, Moscow, 1982), pp. 297–306.

93. K. Kaniuth, Long distance imports in the Bronze Age of Southern Central Asia. *Archäol. Mitt. Iran Turan* **42**, 3–22 (2010).

94. V. I. Sarianidi, Ed., *Margush: Mystery and True of the Great Culture* (Türkmendöwlethabarlary, Ashgabat, 2008).

95. D. Frenez, Manufacturing and trade of Asian elephant ivory in Bronze Age Middle Asia: Evidence from Gonur Depe. *Archaeol. Res. Asia* **15**, 13–33 (2017).

96. D. Frenez, "Torgovlja slonovoj kost'ju iz bivnej Asiatskogo slona" in *Trudy Margianskoj Arkheologicheskoj Ekspeditsii*, N. A. Dubova, Ed. (Staryj Sad, Moscow, 2018), vol. 7, pp. 106–133.

97. V. I. Sarianidi, Ed., *Gonurdepe, Türkmenistan: City of Kings and Gods* (Miras, Ashgabat, 2005).

98. V. I. Sarianidi, N. G. O. Boroffka, N. A. Dubova, Cultural contacts of Margiana, Turkmenistan, in the 3rd millennium BC: New evidence from Gonur Depe, Burial 4150. *Gandhāran Studies* **6**, 1–17 (2012).

99. V. I. Sarianidi, N. G. O. Boroffka, N. A. Dubova, "Kul'turnye kontakty Margiany v III tys. do n.e." in *Trudy Margianskoj Arkheologicheskoj Ekspeditsii*, V. I. Sarianidi, Ed. (Staryj Sad, Moscow, 2014), vol. 4, pp. 127–137.

100. R. N. Spengler, I. de Nigris, B. Cerasetti, M. Carra, L. M. Rouse, The breadth of dietary economy in Bronze Age Central Asia. *J. Archaeol. Sci. Rep.* **22**, 372–381 (2018).

101. B. Lyonnet, N. A. Dubova, Eds., *The World of the Oxus Civilization* (Routledge, London, 2020).

102. R. M. Sataev, N. A. Dubova, M. A. Mamedov, "The internal chronology and periodization of Gonur Depe" in *Antiquities of East Europe, South Asia and South Siberia in the Context of Connections and Interactions within the Eurasian Cultural Space (New Data and Concepts): Proceedings of the International Conference* (IIMK RAS, 2019), 41 pp.

103. O. Lecomte, "Ulug-depe: 4000 years of evolution between plain and desert" in *Historical and Cultural Sites of Turkmenistan* (Turkmen State Publishing Service, Ashgabat, 2011), pp. 223.

104. R. Besenval, A. I. Isakov, Sarazm et les débuts du peuplement agricole dans la région de Samarkand. *Arts Asiat.* **44**, 5–20 (1989).

105. P. L. Kohl, C. C. Lamberg-Karlovsky, R. Maddin, Metallurgical analysis from Sarazm, Tadjikistan SSR. *Archaeometry* **29**, 90–102 (1987).

106. F. T. Hiebert, K. Kurbansakhatov, Eds., *A Central Asian Village at the Dawn of Civilization: Excavations at Anau, Turkmenistan* (Univ. Pennsylvania Museum of Archaeology and Anthropology, Philadelphia, PA, 2003).

107. P. L. Kohl, The Namazga civilization: An overview. *Sov. Anthropol. Archaeol.* **19**, vii–xxxviii (1981).

108. E. E. Kuzmina, *The Origin of the Indo-Iranians*, J. P. Mallory, Ed. (Brill, Leiden, 2007).

109. A. I. Isakov, Sarazm: An agricultural center of ancient Sogdiana. *Bull. Asia Inst.* **8**, 1–12 (1994).
