# Supplementary material for: Silkworm cultivation predating the Silk Road in southern Central Asia (2000 BCE)
Source: Sci Adv. 2026 Jul 23;12(30):eaec8738. doi: 10.1126/sciadv.aec8738 (PMC13394462; doi:10.1126/sciadv.aec8738)
Supplement: Supplementary file 2 [file sciadv.aec8738.v1.pdf]

ARCHAEOLOGY

Silkworm cultivation predating the Silk Road in southern Central Asia (2000 BCE)

Xinying Zhou[1,2,3]*†, Hui Shen[1,2]†, Zhiqing Li[4]†, Robert N. Spengler[5], Guanhan Chen[1,2], Huiyun Rao[1,2], Mutalibjon Khasannov[6,7], Keliang Zhao[1,2], Yan Wu[1,2], Jian Ma[3], Jianxin Wang[3], Akhmadali A. Askarov[6], Farhod Maksudov[6], Qingyou Xia[4]*, Xinyi Liu[8]*, Xiaoqiang Li[1,2]

Copyright © 2026 The Authors, some rights reserved; exclusive licensee American Association for the Advancement of Science. No claim to original U.S. Government Works. Distributed under a Creative Commons Attribution License 4.0 (CC BY).

Historical narratives hold that sericulture originated in China and spread along the Silk Road roughly two millennia ago. In questioning this assumption, we conducted micromorphological and proteomics analyses on three cocoons recovered from the Sapalli Tepe (approximately 2000 to 1500 BCE) site in Uzbekistan. We identified them as *Bombyx mori*, directly dated to 1940 to 1765 cal. BCE, representing the earliest remains of *Bombyx* cocoons. We further report the earliest mulberry (*Morus* sp.) charcoal fragments from Central Asia, attesting to mulberry-*Bombyx* sericulture in the Oxus nearly 2000 years earlier than previously documented. We highlight that early silkworms and mulberry trees may have been introduced to Central Asia along the southern slope of the Himalayas, although other routes could also plausibly explain the observed pattern. This multidisciplinary research rewrites the enigmatic history of early sericulture and its globalization.

INTRODUCTION

Mulberry-*Bombyx* sericulture, the integrated practice of *Bombyx mori* silkworm rearing and mulberry cultivation, has been historically important to cultures across Asia. Genomic and archaeological evidence suggests that *B. mori* was domesticated from its wild progenitor, *Bombyx mandarina*, and its early use has been traced back to 8500 to 5000 years ago in ancient China (*1*–*4*). As the obligate food source for *Bombyx* silkworms, mulberry trees, notably the white mulberry (*Morus alba*), were globally dispersed in antiquity (*5*), but when and how this process of dispersal unfolded remain obscure. It is generally accepted that silk products and their accompanying knowledge spread from ancient China to Central Asia and beyond within the context of the Silk Road, beginning in the late second century BCE, as it is commonly assumed (*4*). However, the conventional timeline of the Silk Road and of Eurasian interconnectivity has been challenged by recent evidence documenting the spread of cereals, fruits, metallurgy, and livestock connecting ancient China with the Central and South Asian environments during the third and second millennia BCE (*6*–*8*). The possibility of an earlier dispersal of silk and sericulture, predating the second century BCE, remains unexplored because of a lack of archaeological evidence.

Today, silk products are mainly manufactured on the basis of mulberry-*Bombyx* sericulture. While silk textile production has long been considered a practice unique to ancient China, emerging evidence suggests that there could have been multiple sericulture traditions using different cocoon-forming insects in antiquity (*9*). People in South Asia developed distinct silk traditions involving non-*Bombyx* silk, notably Tussar (*Antheraea*) and Eri (*Samia*), among other types, such as Muga (*Machilus* or *Litsea*) (*10*). One expert working at Harappan sites in the Indus claimed for the use of non-*Bombyx* (possibly Tussar) silk dating back to at least 2450 to 2000 BCE (*9*, *11*). In addition, a separate Mediterranean origin of silk production using moths of *Pachypasa otus* has been proposed as a possible explanation for references to silk in ancient Greece (*12*, *13*). Other claims for pre-Han (before 202 BCE) silks have been reported from several European and African sites (*14*). However, most of these purported pre-Han silk occurrences outside China have been challenged on methodological grounds or remain subject to further scrutiny (table S1) (*15*, *16*).

During the Han-Roman period, convincing evidence of silk and silk-like fabrics occurs widely across Eurasia (*9*, *17*, *18*). Some of these textiles exhibited production techniques and decorative patterns clearly distinct from those of traditional Chinese silk and were manufactured using cocoons from moths other than *Bombyx*. Many of these other insects likely represent additional attempts at cultivation involving non-*Bombyx* taxa (e.g., Bombycidae, Saturniidae, and Lasiocampidae) that relied on hosts other than mulberry (*17*, *19*). For example, Tussar moth larvae feed on two *Terminalia* species and the sal tree (*Shorea robusta*) (*10*). Together, this evidence depicts an ancient world characterized by diverse sericultural traditions independent of the mulberry-*Bombyx* system, whose relationships to the Chinese tradition remain open inquiry.

It is therefore timely to consider archaeological evidence of silk production outside China, in order to explore regional sericultural traditions and their historical connections, and to critically reassess the Silk Road timeline. Here, we report the discovery of three *B. mori* cocoons, which are directly dated to 1940 to 1765 cal. BCE, associated with abundant mulberry charcoal, together with previously recovered silk-like fabrics from the Sapalli Tepe site in southern Uzbekistan. Our proteomics results single out domesticated silkworms (*B. mori*) from its wild progenitor (*B. mandarina*) and other non-*Bombyx* taxa, while archaeobotanical results indicate the presence of mulberry. This suggests that sericulture involving the *B. mori* caterpillar and the mulberry tree had dispersed from East Asia and developed into a

[1]Institute of Vertebrate Paleontology and Paleoanthropology, Chinese Academy of Sciences, Beijing, China. [2]University of Chinese Academy of Sciences, Beijing, China. [3]Center for Archaeological Cooperation Research along the Silk Road, Northwest University, Xi'an, China. [4]Integrative Science Center of Germplasm Creation in Western China (CHONGQING) Science City & Biological Science Research Center, Southwest University, Chongqing, China. [5]Domestication and Anthropogenic Evolution Research Group, Max Planck Institute of Geoanthropology, Jena, Germany. [6]National Center of Archeology, Academy of Sciences of the Republic of Uzbekistan, Tashkent, Uzbekistan. [7]Faculty of Humanities, Fergana State University, Fergana, Uzbekistan. [8]Department of Anthropology, Washington University in St. Louis, St. Louis, MO, USA.
*Corresponding author. Email: zhouxinying@ivpp.ac.cn (X.Z.); xiaqy@swu.edu.cn (Q.X.); liuxinyi@wustl.edu (X.L.).
†These authors contributed equally to this work.

mulberry-*Bombyx* silk manufacture in southern Central Asia as early as the second millennium BCE.

RESULTS

Sites and chronology

During excavation seasons between 2017 and 2019, a Chinese and Uzbek research team conducted targeted excavations at three Bronze Age sites situated along the Surkhan-Darya River, including Sapalli Tepe (37°27′1.63″N, 66°50′42.19″E), Djarkutan (37°37′57.59″N, 66°57′36.49″E), and Molleli (38°3′56.75″N, 67°42′2.47″E) (Fig. 1 and figs. S1 and S2). All three archaeological sites belong to the Sapalli Culture, which is generally considered as a subdivision of the Bactria-Margiana Archaeological Complex (BMAC) (*20*), also known as the Oxus Civilization (*21*). The BMAC emerged in southern Central Asia in the third millennium BCE, representing a culturally integrated entity characterized by proto-urban settings and agropastoral economies. The Sapalli Culture can be divided into four phases on the basis of respective type sites—three of which are under current investigation—Sapalli, Djarkutan, Kuzalinksi, and Molleli (*22*, *23*). Its material culture shows a close relationship with a variety of predecessor cultures, Namazga-Tepe V and VI in Turkmenistan, Mundigak IV in Afghanistan, and Hissar III and Shahri Sukhtah in Iran (fig. S3 and table S2).

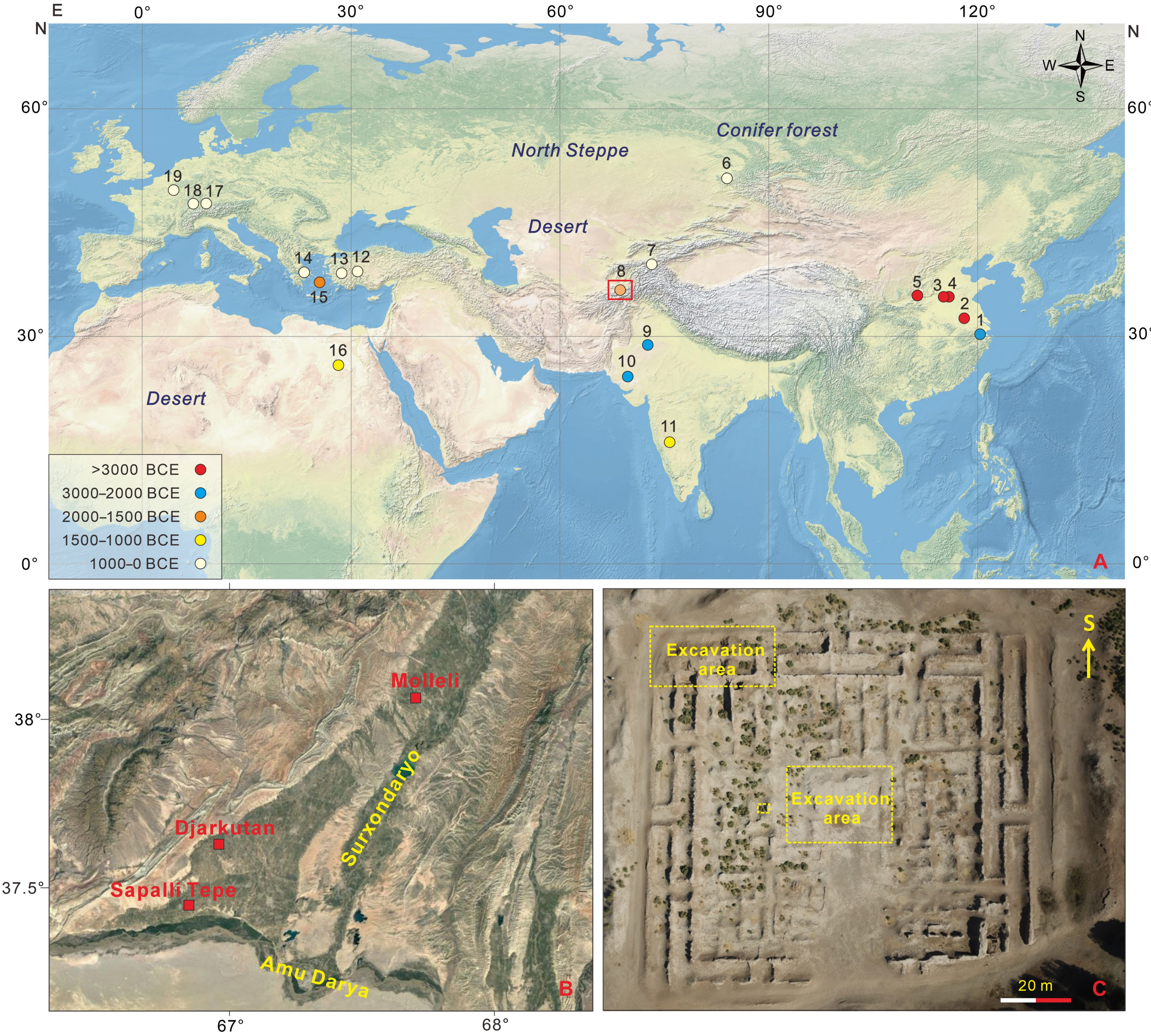

Fig. 1. Location of sites where pre-Han silk has been reported in the Old World. Note that this map includes cases where the identification of silk has been challenged or refuted (all details are listed in Supplementary Text and table S1). (**A**) 1: Qiansanyang; 2: Jiahu; 3: Wanggou; 4: Qingtai; 5: Xiyincun; 6: Pazyryk; 7: Quman; 8: Sapalli Tepe; 9: Harrapa; 10: Chanudaro; 11: Nevasa; 12: Gordion; 13: Sardis; 14: Kerameikos; 15: Akrotiri; 16: Deir al Medina; 17: Hohmichele; 18: Hochdorf; 19: Altrie. (**B**) Map of the Surxondaryo and study sites within this research project. (**C**) Photos (by Dajiang Mavic) of the excavation area of Sapalli Tepe. PHOTO CREDIT: X. ZHOU/IVPP, CAS.

Sapalli Tepe is a well-known site representing one of the oldest BMAC localities in the Sukhan-Darya basin (*24*–*26*). It is located about 8 km from the northern bank of the Amu Darya (fig. S1A). The site was first excavated by the Archaeological Institute of the Uzbek Academy of Sciences between 1969 and 1973 (fig. S4). Its main structure resembles a square fortress or stronghold, with each side measuring ~82 m (fig. S2A). The perimeter is enclosed by mudbrick walls, and this layout is commonly reported from sites during this period, for example, at Sarazm in Tajikistan (*27*). Most of the flotation samples from Sapalli Tepe were collected from excavation areas 1, 2, and 3, which are located between the outer and inner walls of the central fortress (fig. S2, D to F).

The Djarkutan site is located ~60 km north of the modern city of Termez, near a dried-up tributary of the Amu Darya (fig. S1B). As the largest Bronze Age urban settlement in the region, it likely served as a socioeconomic center of the BMAC (*23*, *28*). Covering an extensive area of more than 100 ha, the site lacks clear evidence of defensive enclosures. Its northern and western boundaries are distinguished by hills and gullies, while the eastern side gradually flattens without clear demarcation. The structure interpreted as a palace is situated on a hill at the north-westernmost part of the site and contains a palatial complex (*28*, *29*). To the west of this complex, there is a depression that may have been used as a well or for drainage, which was later filled with ash (fig. S2G). Our samples were mainly collected from the loose fill of this ash pit near the ancient palace.

Molleli is a small Late Bronze Age site covering an area of about 10 ha and located to the northeast of the Sapalli site. Archaeological studies commonly consider the cultural characteristics of Molleli as representing the latest phase of the Sapalli Culture, and it is characterized by material traditions independent from both the Sapalli and Djarkutan featuring distinct pottery typologies and burial practices (*22*, *23*). A section was excavated in 2017 on the playground of a local primary school, and we cleaned the exposed section and collected three flotation samples (fig. S2H).

In this study, we obtained 10 accelerator mass spectrometry ^{14}C measurements from these sites, as presented in the Supplementary Materials. The results align with prior estimates, indicating that Sapalli Tepe was occupied between 2140 and 1800 cal. BCE, Djarkutan between 2140 and 1450 cal. BCE, and Molleli from approximately 1500 cal. BCE (table S3 and fig. S5). One of the three cocoons from Sapalli Tepe is directly dated to 1940 to 1765 cal. BCE, suggesting that the specimens were recovered in situ. Given the shared archaeological context of the three cocoons (mo. T3-1) and the stratigraphic coherence of associated radiocarbon dates, a single direct date appears sufficient to confirm their antiquity.

Silkworm cocoons

The three desiccated cocoons were recovered alongside other charred and desiccated macrobotanical remains during the flotation program. A total of 63 flotation samples were collected from eight contexts at Sapalli Tepe, five at Djarkutan, and three at Molleli (fig. S2). All three cocoons (Sapalli-C-1-3) were recovered from sample no. T3-1 in Trench 3 at Sapalli Tepe. This trench is located in Room 25 (*24*), which has an elongated rectangular shape (dimensions of 3 by 9 m), and was filled with uniquely loose and rich ash.

These ancient cocoons are morphologically like *B. mandarina* and notable smaller than those of domesticated *B. mori* (Fig. 2 and figs. S6 and S7). Scanning electron microscopy (SEM) revealed that the fibers of the ancient cocoons exhibited a similar structure to those of *Bombyx* cocoons, although the fibers were thinner than either modern *B. mori* or *B. mandarina* (Fig. 2). The difference in size may be partially attributed to the desiccation and other posttaphonomic effects, including protein degradation.

Protein profiling analysis using liquid chromatography–tandem mass spectrometry (LC-MS/MS) was performed on all three specimens to determine the species. Silk proteins (fibroin heavy chain, fibroin light chain, fibroin p25, sericin 1, sericin 2, and sericin 3) from a range of species, including *B. mori*, *B. mandarina*, *Antheraea pernyi*, *Antheraea mylitta*, *Antheraea yamamai*, *Antheraea assamensis*, *Samia ricini*, and others (see the Supplementary Materials), were downloaded from the UniProtKB database for peptide identification. Notably, a large number of silk peptides uniquely characteristic of *B. mori* and *B. mandarina* were identified in one specimen (Sapalli-C-1), and those specific to *B. mori* were detected in Sapalli-C-2 but not in Sapalli-C-3 (table S4). These features are characteristic of silkworm cocoons.

To further verify the proteins identified, we compared the LC-MS/MS results against the integrated silkworm proteome databases from National Center for Biotechnology Information and SilkDB. Fibroin heavy chain and sericin 1 were detected in Sapalli-C-1, and sericin 1 was identified in Sapalli-C-2 (Fig. 3 and table S4). Despite a lack of silk proteins in Sapalli-C-3 (table S4), this specimen contained abundant (n = 30,000) proteins (Bmlp1, Bmlp2, and Bmlp6) and storage proteins (SP1 and SP2), which were also present in Sapalli-C-1 and Sapalli-C-2. Collectively, the proteomic profile aligns exclusively with the reference spectra of *B. mori*, confirming an affinity to domesticated mulberry silkworms. We acknowledge that the specimens are morphologically similar to *B. mandarina* and may represent morphological variation in the early domesticated *Bombyx* silkworm. To be clear, it is improbable that any extant non-*Bombyx* taxa not represented in the UniProtKB database could have produced these proteins, as the database covers most species historically used in the various forms of sericulture, none of which provide matching profiles.

Plant remains

We also identified 1244 wood charcoal fragments from floated archaeological samples, which we classified into 10 taxa on the basis of the published literature and our own comparative collection of wood samples (figs. S8 to S11). Cultivated and non-native trees in the assemblage included *Morus* sp. (fig. S8), *Citrus* sp. (fig. S9), and *Ficus* sp. (fig. S10). It is notable that our *Morus* specimens appear morphologically more similar to Chinese white mulberry *M. alba* than black mulberry *Morus nigra* (*30*). Both of them are widely spread throughout Central Asia today, and we acknowledge the lack of published reference for Indian species, such as *Morus indica* and *Morus laevigata*—although neither of which are present in Central Asia today. *Morus* charcoal fragments occurred in sediments from all three sites and showed an increasing trend over time, from 10% at Sapalli Tepe to more than 60% at Molleli (Fig. 4). The percentage of *Citrus* wood is much lower. While ancient *Citrus* remains have never been identified in Central Asian archaeological sites prior, it is well known that citron was cultivated at this period further south (*31*). *Ficus* charcoal only occurred in one sample from Djarkutan. We also identified Maloideae fruit wood remains, as well as riparian species, like *Salix*, *Populus*, and *Tamarix*, and drought-tolerant plants in the Amaranthaceae and Lamiaceae families. Along with the wood charcoal remains, a large assemblage of seeds and fruit fragments were counted and identified (figs. S12 and S13). Cereals included barley (both six-rowed hulled and naked varieties), common wheat, foxtail millet, broomcorn millet, and lentil.

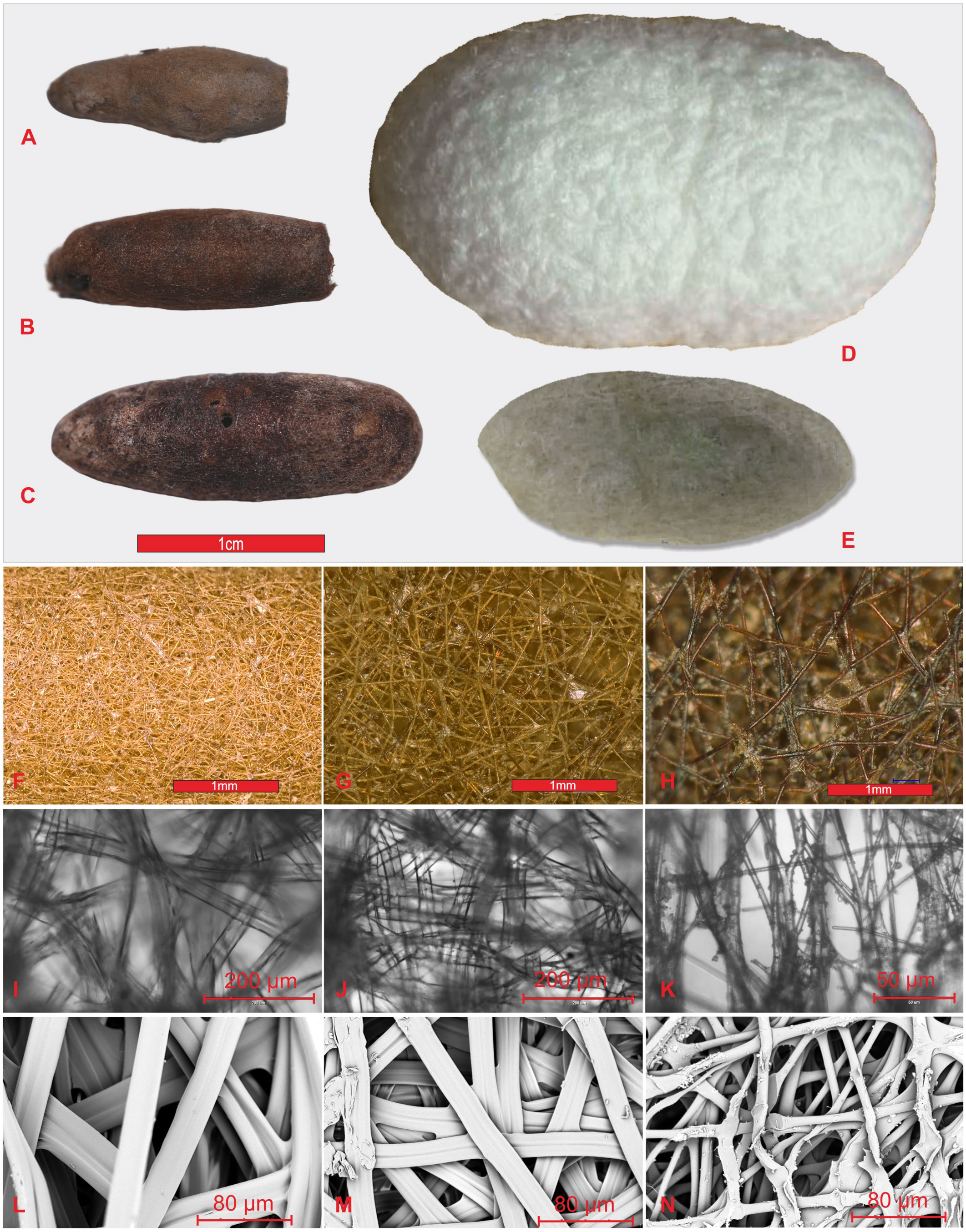

Fig. 2. Comparison between ancient silkworm cocoons unearthed from the Sapalli Tepe site, modern *B. mori*, and wild *B. mandarina*. (**A** to **C**) Photos of cocoons (nos. Sapalli-C-1 to Sapalli-C-3) (from flotation sample no. T3-1). (**D**) Domesticated *B. mori*. (**E**) Wild *B. mandarina*. (**F** to **N**) Stereo microscope, light microscopy and SEM photos of silk fiber. [(F), (I) and (L)] Domesticated *B. mori* silk. [(G), (J) and (M)] Wild *B. mandarina* silk. [(H), (K) and (N)] Ancient cocoon silk. PHOTO CREDIT: X. ZHOU/IVPP, CAS.

Fruit and fiber seeds included plum, Russian olive, grape, pistachio, apple, and flax.

DISCUSSION

Mulberry sericulture in the BMAC

The domestication of *Bombyx* silkworms and the invention of degumming techniques are usually attributed to China (*9*, *32*). Archaeologically preserved *Bombyx* silk fibers have been reported from China dating back to at least 5000 years ago, if not earlier (*2*, *4*, *33*). The archaeological evidence and the close association between *Bombyx* silkworms and white mulberry trees, in ecological terms, point to eastern China as the origin of silkworm domestication while also highlighting the relevance of a narrow belt along the southern slope of the Himalaya, where mulberry trees are abundant. This narrow belt includes northern India, Assam, and Bengal, and

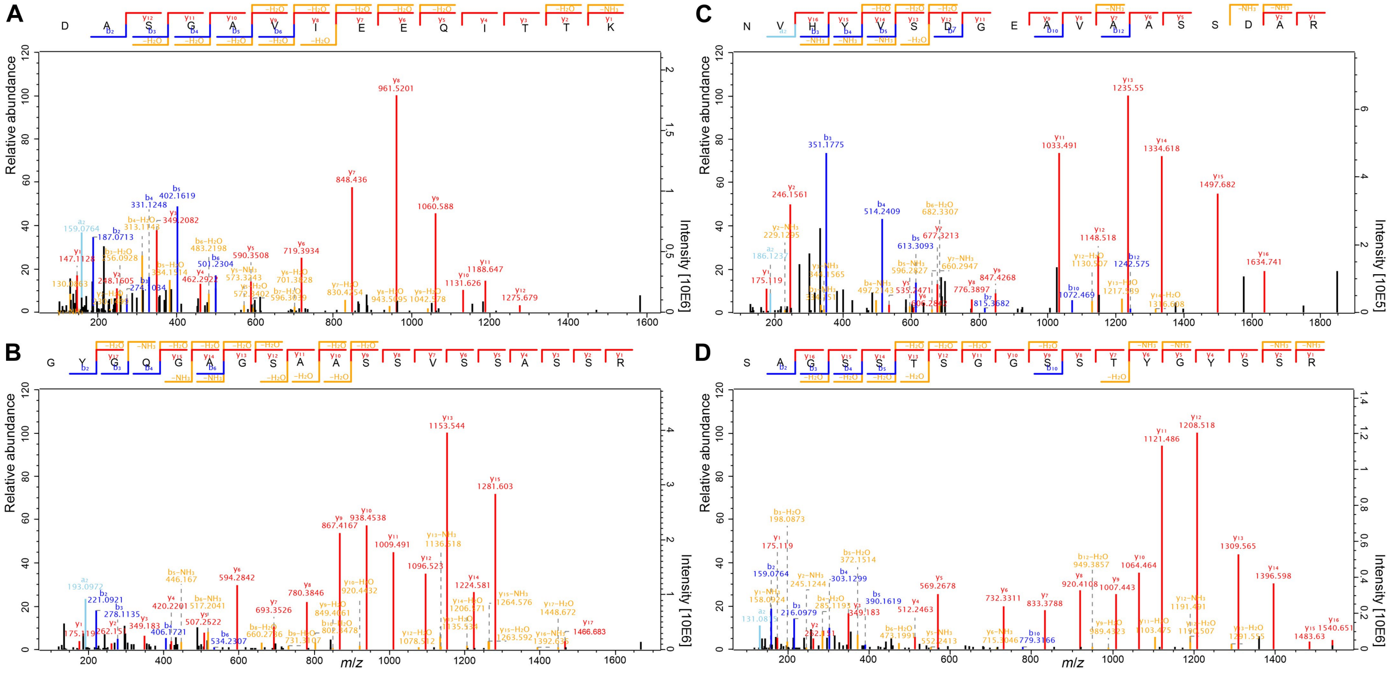

Fig. 3. LC-MS/MS spectra of fibroin heavy chain and sericin 1 in cocoons from Sapalli Tepe. (**A**) Peptide DASGAVIEEQITTK and (**B**) peptide GYGQGAGSAASSVSSASSR of fibroin heavy chain from Sapalli-C-1. (**C**) Peptide NVHYVSDGEAVAASSDAR and (**D**) peptide SAGSSTSGGSSTYGYSSR of sericin 1 from Sapalli-C-2. *m/z*, mass/charge ratio.

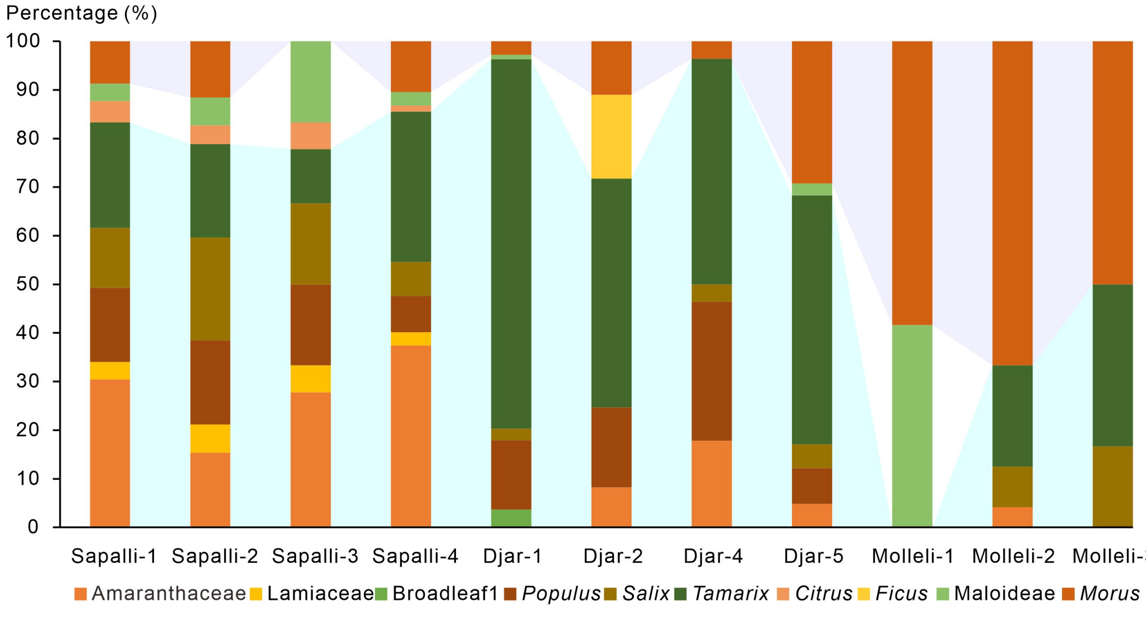

Fig. 4. Assemblage of wood charcoal remains from the three sites: Sapalli, Djarkutan (=Djar), and Molleli. The background shadow of light purple represents the total portion of mulberry wood, and green represents all nonfruit trees.

notably, these regions are historically associated with non-*Bombyx* silk productions. As noted above, the Indus environment has been proposed as a likely original homeland for alternative sericultural traditions—Tussar, Eri, and Muga—using non-*Bombyx* moths. Their antiquity, however, is unknown. One account claims that Tussar silk could date back to the Harappan period (*11*).

Turning to the early globalization of Chinese sericulture, it is generally believed that the *Bombyx* silk tradition spread from China to Central Asia during the Han Dynasty (202 BCE to 220 CE). This historical narrative suggests that a political envoy encouraged transnational exchange of silk alongside other commodities, such as metal coins, woolen materials, and ceramics, marking the beginning of the Silk Road. This proposition has been further reinforced by archaeological finds in northwestern China, such as silk fabrics recovered from Niya and Loulan dating to the time (*4*, *34*). A widely cited alternative narrative suggests that silk was introduced to Hotan (Hetian)

in Xinjiang through the marriage of a Chinese princess to a local prince in the first century CE (sometimes dated to 440 CE) (*4*, *35*). However, our discovery of *B. mori* cocoons at Sapalli Tepe and mulberry charcoal remains at three contemporaneous sites offers a substantially earlier timeline for the dispersal of Chinese sericulture. These findings reveal that Central Asian communities practiced silkworm rearing and mulberry cultivation long before the Han Dynasty, pushing mulberry-*Bombyx* sericulture in this region back in time by nearly 2000 years.

Scholarly interests have recently been drawn to earlier episodes of trans-Eurasian exchange substantially predating the Silk Road, featuring the movements of domesticated plants and animals (*6*, *7*). This process occurred during the third and second millennia BCE and introduced millets of East Asian origins to Central Asia and wheat, barley, sheep, goats, and cattle from West Asia to the regions of modern China. Our identification of *B. mori* cocoons and *Morus* charcoal provides previously unrecognized evidence to place the western expansion of Chinese sericulture within this early episode of "globalization."

This observation is also supported by the co-occurrence of both broomcorn and foxtail millets in our study sites—providing one of the oldest pieces of evidence for foxtail millet in Central Asia. Both millet species were introduced to Central Asia from East Asia during the third and second millennia BCE. Meanwhile, the abundance of mulberry charcoal at all three sedentary sites, associated with the presence of other economically important fruit trees, most newly introduced to the region, such as figs, citrus, and grapes, suggests a cosmopolitan arboriculture involving management of non-native tree crops around the proto-urban center, of which mulberry cultivation was a part. The coexistence of mulberry tree and *Bombyx* silkworm implies localized silk production in the context of a cosmopolitan agro-arboriacultural system in the second millennium BCE.

Additional evidence for local silk production derives from a fabric fragment recovered from Sapalli Tepe during excavations in the 1970s (fig. S4). Although direct analysis was not possible because of conservation restrictions at the National Museum in Tashkent, archival documentation confirms its production via a traditional plain-weave technique of spun yarn, consistent with Chinese style of silk manufacture (fig. S4) (*24*). In addition, rich assortments of textile production tools, including terracotta and stone spindle whorls and needles, are frequently recovered from BMAC sites (*24*, *36*). While spindle whorls were likely also used for wool production, their abundance nonetheless indicates the prominence of textile manufacture at these sites. Another potential source for textile making may have been flax, as flax seeds were also recovered at Sapalli, whereas cotton would not be introduced to the region for another millennium and a half, and hemp would not become a common fiber source in Central Asia during prehistory (*7*, *37*).

Mulberry trees in the BMAC economy

Our finding of mulberry charcoal constitutes the earliest archaeological evidence of *Morus* in Central Asia, predating the previous oldest record of mineralized seeds from medieval cesspits (*38*). The lack of earlier evidence is likely due to archaeobotanical research focusing on seed remains or assemblage formation issues, given that berries are often consumed fresh and do not undergo charring. We address this gap in the data with our charcoal analysis. As mentioned, the abundance of mulberry charcoals highlights the essential role of mulberry trees in the BMAC arboriculture system. This ancient horticulture practice appears to have left a clear legacy: In modern Uzbekistan, mulberry trees continue to be widely cultivated and are designated with important cultural meanings that extend well beyond sericulture and trade.

The *Morus* genus includes 17 species, 13 of which are endemic to East or South Asia, 3 in the Americas, and 1 in West Asia (*5*, *39*). This biogeography of *Morus* trees seems to suggest a natural distribution that did not initially include Central Asia nor the northern fringes of the Tibetan Plateau. Mulberry charcoal does not appear in archaeological sites from Europe or Southwest Asia until the first millennium CE, a process that is thought to be associated with Arab Agricultural Revolution (*40*, *41*). Our mulberry charcoal records recovered from three nearly contemporary sites within the BMAC contexts thus represent some of the earliest evidence for the cultivation and intentional dispersal of mulberry trees into Central Asia.

The co-occurrence of *B. mori* cocoons, *Morus* charcoal, and silk-like fabrics at Sapalli Tepe indicates an unambiguous connection with East Asian sericultural traditions. Given that we provide only a single data point for such sericulture outside eastern China, it is conceptually challenging to verify the precise dispersal process. Nonetheless, we draw the reader's attention to a possible dispersal via a southern route—in contrast to the well-documented northern Silk Road via the Hexi-Tianshan and Inner Asian Mountain Corridors—along the southern Himalayan slopes. Mulberry charcoal has been identified from Mature Harappan contexts dating to before 2000 BCE (*42*, *43*) and in Kashmir Valley by approximately 500 CE (*44*). The Harappan record contrasts to the relative lateness of the *Morus* remains along the northern fringe of the Tibetan Plateau despite this northern route's well-established role in Silk Road narratives. In the Hexi Corridor, mulberry remains have been recovered from Yingshuwo (1500 to 1300 BCE) and Sanjiaocheng (1000 to 400 BCE) (*45*), at least 500 years later than the Harappan and BMAC specimens. There have been no reports of mulberry cultivation along the Inner Asian Mountain Corridor, including the Tianshan foothills, predating 1200 BCE (*46*, *47*). On the basis of current evidence, mulberry records along the northern fringes of the Tibetan Plateau postdate those from the southern fringe of the plateau as well as those associated with the BMAC, presented in this research. Collectively, these data infer a connection between the Sapalli Tepe evidence and South Asian–related dispersal, although future investigations—particularly those differentiating Chinese white mulberry from Indian mulberry at the species level—will be essential for clarifying the situation.

The recovery of *Citrus* wood charcoal remains at Sapalli Tepe also signifies a close relationship with South Asia. As a typical thermophilic and hygrophilic plant, the genus *Citrus* originated in the southeastern foothills of the Himalayas and south-central China (*48*, *49*), and citron cultivation has been suggested for the Mature Harappan phase (*42*). Previous studies have speculated about a southern dispersal route via the Himalayan slopes and the southern Tibetan Plateau, perhaps operating in both directions (*50*–*52*). Notably, such a southern connection is consistent with intensive material exchanges between the BMAC and the Indus, which have been heavily discussed in the literature for more than a century (*53*–*55*).

We acknowledge that the hypothesis featuring the southern route of mulberry-*Bombyx* dispersal is complicated by claims that the Harappan silk tradition was based on non-*Bombyx* taxa and the historical continuity of Tussar and Eri sericulture in South Asia. It is also not implausible that *B. mori* was introduced to the BMAC via a northern route—along the Hexi and Inner Asian Mountain Corridors.

Given the single data point we contribute to the map of silk dispersal, all theories remain subject to future scrutiny. It is useful, however, to highlight the plausibility of a southern Himalayan connection, as this is often overlooked in scholarly discussions of both the initial formation of the Silk Road and the trans-Eurasian exchange of prehistoric foodways.

Collectively, communities inhabiting proto-urban centers across the northern zone of the Oxus cultural sphere (BMAC) during the second millennium BCE demonstrated knowledge of sericulture. This is evidenced by the co-occurrence of *B. mori* silkworm cocoons, together with mulberry (*Maru* cf. *alba*) charcoal, and silk fabric fragments, confirming silk production in southern Central Asia by approximately 2000 BCE. While the cocoons are morphologically similar to wild forms, protein fingerprinting analyses place them closer to the domesticated silkworm (*B. mori*) rather than the progenitor (*B. mandarina*) or another cocoon-forming species. Our data establish the earliest documented mulberry-*Bombyx* sericulture outside ancient China, situating the dispersal of this technology in an early episode of globalization. We highlight a plausible dispersal route following the southern slope of the Himalayas before reaching Central Asia, potentially fostered by cultural connections with the Indus during the second millennium BCE, although other possibilities remain. The adoption of citron trees and foxtail millet further underscores the cosmopolitan nature of the BMAC economy and its role in early globalization. The identification of Chinese sericulture in the BMAC challenges the conventional chronology of the Silk Road, predating the previously accepted timeline by nearly two millennia. Our results also raise the question whether multiple forms of sericulture were widespread across Asia during the second/third millennia BCE than has been previously recognized. Future research will no doubt deepen our understanding by clarifying the continuity of the mulberry-*Bombyx* knowledge in Central Asia after its initial introduction, the potential routes of its dispersal, and the regional diversities of silk production practices in deep antiquity.

MATERIALS AND METHODS

Experimental design

We performed a comprehensive analysis of the flotation materials, including silkworm cocoons, wood charcoal, and plant seeds, collected from three BMAC sites in southern Uzbekistan. By identifying these remains, we aimed to investigate the nature of the cocoons and assess whether silk production knowledge had reached Central Asia at this early stage. Regarding the cocoons, we used morphological observation, protein analysis, and radiocarbon dating to determine their species and dates. Through anatomical identification of the wood charcoal and seeds, we try to figure out the woods and crops exploited by local populations. Integrating these findings, our study reveals the presence of domesticated *Bombyx* silkworms, mulberry trees, and other fruits and crops at nearly 2000 BCE, suggesting that a *Bombyx* sericulture system had existed in southern Central Asia long before the establishment of the historical Silk Road.

Excavations and sampling

Most of the flotation samples from Sapalli Tepe were collected from excavation areas 1, 2, and 3, which are located between the outer and inner walls of the central fortress of the site (fig. S2, D to F). There is about 50 cm of sediment covering soil above the cultural layers in these areas. After clearing and exposing these overlaying strata, we noted obvious black organic residues marking the cultural layers in these three areas. Thirty-five flotation samples were collected and assigned a sample number on the basis of the site name and excavation area. At Sapalli Tepe, excavation area 4 has been interpreted by the excavators as a storage pit, and 5, 6, and 7 are located in the central square of the site. A small number of research samples were taken from each area. We sampled an excavation wall of a pit at Djarkutan, located near the road on the western side of an area that the local archaeologists have interpreted to be a palace, where we took six samples. At the Molleli site, a section was excavated in 2017 by the playground of a local primary school, and we cleaned the exposed section and collected three flotation samples (fig. S2H).

All sediment samples were collected during the 2017 excavation season at 5-cm contiguous intervals. The sediment samples were collected for flotation, and at least 30 liters of sediment was processed for each sample. The recovery of archaeobotanical material at Sapalli was aided by the use of an overflow tank machine, which facilitated rapid processing. All samples were floated using a 0.3-mm mesh screen. All of the light fraction portions were taken to the archaeobotany laboratory of the Institute of Vertebrate Paleontology and Paleoanthropology (IVPP) of the Chinese Academy of Sciences. The light fraction of the archaeobotanical samples was first sorted, weighed, and then identified. We analyzed 63 samples from these three Bronze Age sites, of which 60 samples had seeds and fruits. A total of 3295 seeds, fruits, and fragments were counted and recorded.

Radiocarbon dating

Samples for radiocarbon dating were selected from the light fraction of the floated sediments representing different layers of the excavated section. Five dates were obtained from charred cereal grains, including three barley and two wheat grains. The samples were collected during the 2017 excavation season and were recorded and plotted using a Total Station. The samples were collected from two different pits (T0505 and T0103), from the depth interval of 40 to 140 cm below the ground surface. First, they were cleaned of any adhering sediment before being crushed. This was followed by an acid-base-acid chemical pretreatment, which consisted of washing in hot HCl (hydrochloric acid; 5%), rinsing, treatment with 1% NaOH (sodium hydroxide), rinsing again, and then treatment with hot HCl (5%), rinsing, and drying. The pretreated samples were then combusted to CO_2 (carbon dioxide) by oxidation at 800°C using CuO [copper(II) oxide]. The CO_2 was purified in the presence of silver wire to absorb any SO_x and NO_x produced and was then reduced to graphite with H_2 (hydrogen gas) at 550°C using an iron catalyst. The pressed graphite targets were sent to the Radiocarbon Dating Laboratory of Beta Analytic in Miami, US, for accelerator mass spectrometry measurements. The radiocarbon ages and related information, including age calibration performed using the OxCal version 4.2 program44, are provided in table S3 and fig. S5.

Morphology of silkworm cocoons

Cocoon silk fibers were coated with Au (gold) (MCI000, Tokyo, Japan) and observed by SEM (Hitachi SU3500, Tokyo, Japan). Compared to domesticated and wild mulberry silkworm cocoons, our ancient cocoons showed a similar size and shape to wild morphs, as they are considerably smaller than the domesticated cultivars (fig. S6). Specifically, the Sapalli-C-1 specimen is about 1.35 cm in length, and the widest part is 0.53 cm in width. One of the ends has been broken and now has a smooth and straight hole (fig. S7A-1). This was likely

caused by the hatching of the moth. This practice aligns with archaeological evidence from ancient Central Asia, where people typically used hatched cocoons to allow more adult moths to mate and lay eggs (*4*). Hatched cocoons can be used when the silk fibers are spun into yarn, as opposed to being used as a continuous thread. In contrast, cocoons in eastern China were typically preserved intact and processed before the moths emerged, ensuring their suitability for silk reeling. The Sapalli-C-1 cocoon is constructed out of loose silk fibers, which now have a yellowish color (fig. S7, A-2 and A-3). Meanwhile, the silk fibers appear to have pealed in half along their length, a typical form of degradation, as an individual silk fiber is produced by adhering two fibers together. Unlike the specimens of 2 and 3, Sapalli-C-1 contains nearly no adhesive substance, implying that this sample is less degraded than the other two cocoons.

The Sapalli-C-2 specimen also shows a smooth and even hole at one end, similar to the sample of Sapalli-C-1. This cocoon is 1.53 cm in length and 0.54 cm in width. The superficial silk fibers are relatively tight and now look dark yellow. On the surface of the silk fibers, a large amount of adhesive substance was present, providing a shiny quality. This material might be the remains of sericin resulting from long-term degradation.

The Sapalli-C-3 specimen maintained the compete elliptic shape in the cross section, allowing us to confirm that the size is similar to those of wild silkworm cocoons. There are two small holes in the central part of this cocoon, and the diameter of each hole is less than 1 mm (fig. S7C-2). The holes are likely the exit holes of parasitic wasps, which would explain why this cocoon is not hatched. The wasps lay their eggs on the caterpillar, which then builds its cocoon around itself with the developing wasp larva feeding on the caterpillar from the inside. The superficial silk fibers are also tight and thick, covering the whole inner cocoon layer. Compared to the other two cocoons, Sapalli-C-3 is the largest, and its length reaches about 2 cm and the width is 0.69 cm. Unlike the other two cocoons, the Sapalli-C-3 cocoon is dark brown, and some of the silk fibers have even turned black. In addition, the light gray adhesive subsistence is more abundant and is observable on almost all the silk fibers, mostly noticeably where they overlap. All of these features suggest that Sapalli-C-3 exhibits the highest degradation among the three specimens, a conclusion that was also supported by the paleoproteomic results, as the main characteristic proteins of silkworm cocoons, including fibroin heavy chain and sericin 1, were identified in Sapalli-C-1. Sericin 1 was identified in Sapalli-C-2, while none of these proteins were identified in Sapalli-C-3.

Protein analysis

Cocoon silk protein extraction and LC-MS/MS were performed according to published protocols (*56*) at the Biological Science Research Center, Southwest University, China. In summary, ~1 mg of material was excised from each ancient cocoon (processed as individual samples) and dissolved in 0.1 ml of 9 M LiSCN (lithium thiocyanate) for protein extraction. The dissolved proteins were then digested with trypsin. Tryptic peptides were analyzed using a Thermo Fisher Scientific Q Exactive mass spectrometer under the standard parameters. Mass spectrum raw data were analyzed with MaxQuant software (version 1.5.3.17), and protein identification was executed against the silk proteins from the UniProtKB database across a range of different silk genera and species or the integrated silkworm proteome database from National Center for Biotechnology Information and SilkDB. The database includes 57 species, namely *B. mori*, *B. mandarina*, *A. pernyi*, *A. mylitta*, *A. yamamai*, *A. assamensis*, *S. ricini*, *Galleria mellonella*, *Papilio machaon*, *Haritalodes derogate*, *Danaus plexippus*, *Heliothis virescens*, *Leptidea sinapis*, *Pieris macdunnoughi*, *Tineola bisselliella*, *Vanessa tameamea*, *Danaus chrysippus*, *Diatraea saccharalis*, *Pieris brassicae*, *Cnaphalocrocis medinalis*, *Mythimna separata*, *Papilio xuthus*, *Euphydryas editha*, *Leptosia nina*, *Parnassius mnemosyne*, *Corcyra cephalonica*, *Yponomeuta evonymella*, *Ostrinia furnacalis*, *Hyphantria cunea*, *Brenthis ino*, *Deinopis spinosa*, *Cricula trifenestrata*, *Oligotoma nigra*, *Tribolium castaneum*, *Actias selene*, *Hydropsyche angustipennis*, *Vespula maculifrons*, *Verbena squamosa*, *Cardiocondyla obscurior*, *Caerostris darwini*, *Caerostris extrusa*, *Lasius platythorax*, *Odynerus spinipes*, *Bombus terrestris*, *Manduca sexta*, *Arctia plantaginis*, *Vespula pensylvanica*, *V. vulgaris*, *Apis mellifera*, *Bombus bifarius*, *Bombus impatiens*, *Sitophilus oryzae*, *Neodiprion lecontei*, *Papilio machaon*, *Operophtera brumata*, *Habropoda laboriosa*, and *Dinoponera quadriceps*. The identified proteins from each ancient cocoon are shown in table S4.

Anatomical identification of wood charcoal

The anatomical identification of wood charcoal fragments (>4.0 mm) was conducted at the IVPP of the Chinese Academy of Sciences. They were first observed under stereomicroscopy and then photographed under a scanning electron microscope (Zeiss MA EVO25) (figs. S8 to S11) on the basis of the anatomical features shown in three sections (transverse, radial, and tangential) and with reference to published modern wood anatomy references (*57*, *58*). Meanwhile, we also collected modern wood specimens for comparison, as shown in figs. S8 to S11. The collected modern comparative woods include white mulberry (*M. alba*), black mulberry (*M. nigra*), citron (*Citrus medica*), and fig (*Ficus carica*).

In addition to the cultivated or managed species, we also recognized wild species, notably *Salix*, *Populus*, and *Tamarix*, which are all ecologically constrained in their growing conditions to wet areas near riverbanks. Among them, *Tamarix* charcoal fragments were the most abundant and occurred in almost all samples, changing from 21% in Sapalli Tepe and 55% in Djarkutan to 18% in Molleli (Fig. 4 and fig. S11). *Salix* was found at all three sites as well, usually comprising less than 10% of the wood within a sample. *Populus* varied in abundance at Sapalli Tepe and Djarkutan, and it was absent in the sediments from Molleli. Amaranthaceae and Lamiaceae are both diverse families, and many of the species are typically found in dry or saline conditions; hence, we did not attempt subfamilial classifications. While we only have three data points to work with, we cautiously point out a possible temporal transition within the charcoal assemblages from samples dominated by native desert/riparian wood at Sapalli Tepe and Djarkutan to a greater percentage of fruit tree wood, especially *Morus*, at Molleli. The specific description of representative plants is listed as follows.

Morus sp.

The wood charcoal from our study sites shows distinct growth rings (fig. S8A). Vessels are in clusters and radial multiples of two to four; the groups of small vessels along with parenchyma, especially in latewood, often form a pattern of tangential or discontinuous oblique bands. Perforations are simple in horizontal to oblique end walls. Rays are usually one to five cells wide and up to 35 cells high and heterocellular, composed of procumbent, square, irregular, and upright cells. Comparatively, the ancient wood charcoal is more like the white mulberry (fig. S8, B and C), as opposed to the black mulberry (fig. S8D).

Citrus sp.

On the basis of the morphology alone, we have not tried to differentiate between citron and pummelo, and we simply labeled the ancient

wood charcoal as *Citrus* sp. (fig. S9). That said, we assume that it is citron on the basis of the known distribution of the fruit across India at this time. The pores are diffuse, with no clear growth rings (fig. S9A). Vessels are solitary, in radial multiples of two to four or in small clusters of three or four. The parenchyma is obvious, usually vasicentric and confluent in tangential bands. Perforations are simple in oblique end walls. Rays are composed of both uniseriate and multiseriate rays. The uniseriate rays are usually three to eight cells high, and the multiseriate rays are mostly two or three cells wide and 10 to 25 cells high. They are homocellular and consist of only procumbent cells.

Ficus sp.

Growth rings are not overly distinct (fig. S10). Vessels are diffuse, usually in radial multiples of two to six, or solitary, in small clusters of three to five. Parenchyma is evident, in tangential bands that are vasicentric and marginal. Perforations are simple in slightly oblique end walls. Intervessel pits are alternate. Rays are usually two to six cells wide, and the height can reach almost 1 mm, associated with a small number of uniseriate rays. The rays are composed of heterocellular, upright, square, and procumbent cells.

Tamarix sp.

The growth rings are distinct (fig. S11). Vessels are usually solitary, with a small number of clusters and radial multiples of mostly two. Parenchyma is evident and usually vasicentric. Perforations are simple in horizontal end walls, and the intervessel pits are alternate. Rays are much wide and high, up to 15 cells in width and 1 mm in height. The rays in radial sections are heterocellular, usually composed of procumbent cells, with some cells square or weakly upright.

Broadleaf 1

Growth rings of this kind of wood charcoal are distinct (fig. S11). Vessels in the earlywood are much bigger than those in the latewood. Vessels are rounded, solitary, or in radial multiples of two. Parenchyma is abundant, especially in the earlywood, and is usually vasicentric. Perforations are simple in horizontal to oblique end walls. Intervessel pits are alternate. Rays are composed of both uniseriate and multiseriate forms. The uniseriate rays are usually less than four cells high, and the multiseriate rays are mostly two to six cells wide and more than nine cells high. Rays in the radial section are mostly homocellular, composed of procumbent cells, or sometimes heterocellular with a small number of square cells.

Charred and uncarbonized seeds

All the plant seeds from these three Bronze Age sites were identified at the IVPP of Chinese Academy of Sciences, and the total number of charred seeds from 63 samples reached 3295. Specifically, a total of 1497 carbonized seed specimens were identified at Sapalli (fig. S12). The seed remains were predominantly crop seeds, mainly barley and wheat, comprising the following: six-row barley (*Hordeum vulgare* var. *hexastichum*), naked barley (*H. vulgare* var. *nudum*), common wheat (*Triticum aestivum*), broomcorn millet (*Panicum miliaceum*), and lentil (*Vicia lens*). In addition, 29 fragmented cereal remains, 3 legume fragments, and 3 spikelet fragments were found, although these lacked diagnostic features for further identification.

The site also yielded numerous carbonized fruits, nuts, and fiber crops, including the following: plum (*Prunus* sp.), Russian olive (*Elaeagnus angustifolia*), grape (*Vitis vinifera*), pistachio (*Pistacia vera*), and flax (*Linum usitatissimum*). Weed seeds preserved at the site included the following: camelthorn (*Alhagi camelorum*), goosefoot (*Chenopodium* sp.), knotweed (*Persicaria* sp.), sedge (*Cyperus* sp.), false cleavers (*Galium spurium*), and Syrian mustard (*Euclidium syriacum*).

A total of 1691 plant seeds were identified in the sediments from the Djarkutan site, including *H. vulgare*, *H. vulgare* var. *hexastichum*, *H. vulgare* var. *nudum*, *T. aestivum*, *P. miliaceum*, *V. lens*, pea (*Pisum sativum*), grasspea (*Lathyrus sativus*), *V. vinifera*, *Prunus* sp., apple (*Malus* sp.), *E. angustifolia*, *L. usitatissimum*, *A. camelorum*, *G. spurium*, alfalfa (*Medicago* sp.), *Chenopodium* sp., *Cyperus* sp., and *E. syriacum* (fig. S13).

A total of 107 plant seeds were recovered from the Late Bronze Age Molleli site. The identified crop remains include the following: *T. aestivum*, foxtail millet (*Setaria italica*), and *P. miliaceum*. Other plant remains consist primarily of weeds and wild species: *G. spurium*, *Chenopodium* sp., *A. camelorum*, *Cyperus* sp., sorrel (*Rumex acetosa*), and *E. syriacum*.

Statistical analysis

All statistical methods and analyses used in this study are described in the main text and figure legends.

Supplementary Materials

This PDF file includes:
Supplementary Text
Figs. S1 to S13
Tables S1 to S4
References

REFERENCES

1. Q. Xia, Y. Guo, Z. Zhang, D. Li, Z. Xuan, Z. Li, F. Dai, Y. Li, D. Cheng, R. Li, T. Cheng, T. Jiang, C. Becquet, X. Xu, C. Liu, X. Zha, W. Fan, Y. Lin, Y. Shen, L. Jiang, J. Jensen, I. Hellmann, S. Tang, P. Zhao, H. Xu, C. Yu, G. Zhang, J. Li, J. Cao, S. Liu, N. He, Y. Zhou, H. Liu, J. Zhao, C. Ye, Z. Du, G. Pan, A. Zhao, H. Shao, W. Zeng, P. Wu, C. Li, M. Pan, J. Li, X. Yin, D. Li, J. Wang, H. Zheng, W. Wang, X. Zhang, S. Li, H. Yang, C. Lu, R. Nielsen, Z. Zhou, J. Wang, Z. Xiang, J. Wang, Complete resequencing of 40 genomes reveals domestication events and genes in silkworm (*Bombyx*). *Science* **326**, 433–436 (2009).
2. Y. Gong, L. Li, D. Gong, H. Yin, J. Zhang, Biomolecular evidence of silk from 8,500 years ago. *PLOS ONE* **11**, e0168042 (2016).
3. H. Xiang, X. Liu, M. Li, Y. Zhu, L. Wang, Y. Cui, L. Liu, G. Fang, H. Qian, A. Xu, W. Wang, S. Zhan, The evolutionary road from wild moth to domestic silkworm. *Nat. Ecol. Evol.* **2**, 1268–1279 (2018).
4. F. Zhao, Ed., *Silk Journey: Chinese Silk and the Silk Road* (Huangshan Publishing House, Hefei, 2021).
5. C.-X. Yang, S.-Y. Liu, N. J. C. Zerega, G. W. Stul, E. M. Gardner, Q. Tian, W. Gu, Q. Lu, R. A. Folk, H. R. Kates, R. P. Guralnick, D. E. Solti, P. S. Soltis, Y.-H. Wang, T.-S. Yi, Phylogeny and biogeography of *Morus* (Moraceae). *Agronomy* **13**, 2021 (2023).
6. X. Liu, P. J. Jones, G. M. Matuzeviciute, H. V. Hunt, D. L. Lister, T. An, N. Przelomska, C. J. Kneale, Z. Zhao, M. K. Jones, From ecological opportunism to multi-cropping. *Quat. Sci. Rev.* **206**, 21–28 (2019).
7. R. N. Spengler, Ed. *Fruit from the Sands: The Silk Road Origins of the Foods We Eat* (Univ. California Press, Oakland, CA, 2019).
8. X. Zhou, J. Yu, R. N. Spengler, H. Shen, K. Zhao, J. Ge, Y. Bao, J. Liu, Q. Yang, G. Chen, P. W. Jia, X. Li, 5,200-year-old cereal grains from the eastern Altai Mountains redate the trans-Eurasian crop exchange. *Nat. Plants* **6**, 78–87 (2020).
9. I. Good, Archaeological textiles: A review of current research. *Ann. Rev. Anthropol.* **30**, 209–226 (2001).
10. R. S. Peigler, "Wild silks: Their entomological aspects and their textile applications" in *Handbook of Natural Fibres (Second Edition)*, R. M. Kozłowski, M. Mackiewicz-Talarczyk, Eds. (Woodhead Publishing, Cambridge, 2020), pp. 715–745.
11. I. Good, J. M. Kenoyer, R. H. Meadow, New evidence for early silk in the Indus Civilization. *Nat. Prec.* **2008**, 10.1038/npre.2008.1900.1 (2008).
12. G. M. Richter, Silk in Greece. *Am. J. Archaeol.* **33**, 27–33 (1929).
13. I. Good, "The archaeology of early silk" in *Textile Society of America Symposium Proceedings* (Textile Society of America, Millersville, MD, 2002).
14. I. Good, On the question of silk in pre-Han Eurasia. *Antiquity* **69**, 959–968 (1995).

15. L. Bender Jørgensen, The question of prehistoric silks in Europe. *Antiquity* **87**, 581–588 (2013).
16. C. Margariti, S. Protopapas, V. Orphanou, Recent analyses of the excavated textile find from Grave 35 HTR73, Kerameikos cemetery, Athens. *Greece. J. Archaeol. Sci.* **38**, 522–527 (2011).
17. N. K. Adams, W. S. Webb, "Silk in Ancient Nubia: One Road, Many Sources" in *Silk Roads, Other Roads: Textile Society of America 8th Biennial Symposium* (Smith College, Northampton, MA, 2002), pp. 81–91.
18. B. Hildebrandt, The terminology of silks in texts of the Roman Empire: Qualities, origins, products, and uses. *Acta Via Ser.* **6**, 117–139 (2021).
19. R. S. Peigler, Wild silks of the world. *Am. Entomol.* **39**, 151–162 (1993).
20. V. I. Sarianidi, G. Puschnigg, The fortification and palace of northern Gonur. *Iran* **40**, 75–87 (2002).
21. H. P. Francfort, "The early periods of Shortughai (Harappan) and the western Bactrian culture of Dashly" in *South Asian Archaeology 1981* (Cambridge Univ. Press, Cambridge, 1984), pp. 170–175.
22. A. A. Askarov, Southern Uzbekistan in the second millennium D.C. *Sov. Anthropol. Archaeol.* **19**, 256–272 (1981).
23. A. H. Dani, V. M. Masson, Eds., *History of Civilizations of Central Asia. Volume I: The Dawn of Civilization: Earliest Times to 700 B.C.* (UNESCO Publishing, Paris, 1992).
24. A. A. Askarov, Ed., *Sapalli Tepe* (FAN, Tashkent, 1973).
25. A. A. Askarov, Ed., *Drevenezemledel'cheskaja Kul'tura Bronzovogo Veka Juzhnogo Uzbekistana* [The Ancient Bronze Age Farmers' Culture of Southern Uzbekistan] (FAN, Tashkent, 1977).
26. L. P'yankova, Central Asia in the Bronze Age. *Antiquity* **68**, 355–372 (1994).
27. A. Razzokov, Ed., *Sarazm* (Echod, Dushanbe, 2008).
28. A. A. Askarov, B. Abdullaev, Eds., *Djarkutan (K Probleme Protogorodskoj Tsivilizatsii Na Juge Uzbekistana)* [Djarkutan (On the Question of a Proto-Urban Civilization in Southern Uzbekistan)] (FAN, Tashkent, 1983).
29. A. A. Askarov, T. Shirinov, The "palace," temple, and necropolis of Jarkutan. *Bull. Asia Inst.* **8**, 13–25 (1994).
30. E. Karami, K. Pourtahmasi, S. Shahverdi, Wood anatomical structure of *Morus alba* L. and *Morus nigra* L., native to Iran. *Not. Sci. Biol.* **2**, 129–132 (2010).
31. S. Muthukumaran, Ed., *The Tropical Turn: Agricultural Innovation in the Ancient Middle East and the Mediterranean* (Univ. California Press, Oakland, CA, 2023).
32. Z. H. Xiang, J. T. Huang, J. G. Xia, C. Lu, Eds., *Biology of Sericulture* (China Forestry Publishing House, Beijing, 2005).
33. H. Zheng, R. Yang, J. Guo, Z. Xie, J. Liu, H. Yang, L. Jia, J. Yu, Q. Cai, F. Tang, Y. Zhou, Evidence of the use of silk by bronze age civilization for sacrificial purposes in the Yangtze River basin of China. *Sci. Rep.* **14**, 29175 (2024).
34. Z.-Y. Yu, The excavation report of cemetery M8(95MNI) at Niya site in Minfeng County. *Xinjiang. Cult. Relics* **1**, 4–40 (2002).
35. J. E. Hill, Ed. *Through the Jade Gate to Rome: A Study of the Silk Routes during the Later Han Dynasty 1st to 2nd Centuries CE* (BookSurge Publishing, Charleston, SC, 2009), pp. 466–467.
36. V. I. Sarianidi, Temples of Bronze Age Margiana. *Antiquity* **68**, 388–397 (1994).
37. R. N. Spengler, Agriculture in the Central Asian Bronze Age. *J. World Prehist.* **28**, 215–253 (2015).
38. B. Mir-Makhamad, S. Stark, S. Mirzaakhmedov, H. Rahmonov, R. N. Spengler, Food globalization in southern Central Asia: Archaeobotany at Bukhara between antiquity and the Middle Ages. *Archaeol. Anthropol. Sci.* **15**, 124 (2023).
39. A. Tikader, Distribution, diversity, utilization and conservation of mulberry (*Morus* spp.) in North West of India. *Asian Australas. J. Plant Sci. Biotechnol* **5**, 67–72 (2011).
40. A. Livarda, M. van der Veen, Social access and dispersal of condiments in North-West Europe from the Roman to the medieval period. *Veg. Hist. Archaeobotany* **17**, 201–209 (2008).
41. J. M. Marston, L. Castellano, Crop introductions and agricultural change in Anatolia during the long first millennium CE. *Veg. Hist. Archaeobotany* **35**, 129–142 (2026).
42. S. Saraswat, "Banawali (29°37′5″; 75°23′6″), District Hissar" in *Indian Archaeology 1996–1997: A Review* (Archaeological Survey of India, New Delhi, 2002), pp. 203.
43. E. Asouti, D. Q. Fuller, Eds., *Trees and Woodlands of South India: Archaeological Perspectives* (Munshiram Manoharlal Publishers, New Delhi, 2008), p. 126.
44. F. A. Lone, M. Khan, G. M. Buth, Eds., *Palaeoethnobotany: Plants and Ancient Man in Kashmir* (CRC Press, Boca Raton, FL, 1993).
45. H. Shen, X. Zhou, A. Betts, P. W. Jia, K. Zhao, X. Li, Fruit collection and early evidence for horticulture in the Hexi Corridor, NW China, based on charcoal evidence. *Veg. Hist. Archaeobotany* **28**, 187–197 (2019).
46. H. E. Jiang, Ed., *Agricultural Activities and Plant Utilization of the Ancient Yanghai People, Turpan of Xinjiang, China* (Science Press, Beijing, 2022).
47. H. Shen, R. N. Spengler, X. Zhou, A. Betts, P. W. Jia, K. Zhao, X. Li, Seeing the wood for the trees: Active human–environmental interactions in arid northwestern China. *Earth Syst. Sci. Data* **16**, 2483–2499 (2024).
48. G. A. Wu, J. Terol, V. Ibanez, A. López-García, E. Pérez-Román, C. Borredá, C. Domingo, F. R. Tadeo, J. Carbonell-Caballero, R. Alonso, F. Curk, D. Du, P. Ollitrault, M. L. Roose, J. Dopazo, F. G. Gmitter, D. S. Rokhsar, M. Talon, Genomics of the origin and evolution of *Citrus*. *Nature* **554**, 311–316 (2018).
49. Y. Huang, J. He, Y. Xu, W. Zheng, S. Wang, P. Chen, B. Zeng, S. Yang, X. Jiang, Z. Liu, L. Wang, X. Wang, S. Liu, Z. Lu, Z. Liu, H. Yu, J. Yue, J. Gao, X. Zhou, C. Long, X. Zeng, Y.-J. Guo, W.-F. Zhang, Z. Xie, C. Li, Z. Ma, W. Jiao, F. Zhang, R. M. Larkin, R. R. Krueger, M. W. Smith, R. Ming, X. Deng, Q. Xu, Pangenome analysis provides insight into the evolution of the orange subfamily and a key gene for citric acid accumulation in citrus fruits. *Nat. Genet.* **55**, 1964–1975 (2023).
50. C. J. Stevens, C. Murphy, R. Roberts, L. Lucas, F. Silva, D. Q. Fuller, Between China and South Asia: A Middle Asian corridor. *Holocene* **26**, 1541–1555 (2016).
51. D. Q. Fuller, C. J. Stevens, Between domestication and civilization: The role of agriculture and arboriculture in the emergence of the first urban societies. *Veg. Hist. Archaeobotany* **28**, 263–282 (2019).
52. X. Liu, D. L. Lister, Z. Zhao, C. A. Petrie, X. Zeng, P. J. Jones, R. A. Staff, A. K. Pokharia, J. Bates, R. N. Singh, S. A. Weber, G. M. Matuzeviciute, G. Dong, H. Li, H. Lü, H. Jiang, J. Wang, J. Ma, D. Tian, G. Jin, L. Zhou, X. Wu, M. K. Jones, Journey to the east: Diverse routes and variable flowering times for wheat and barley en route to prehistoric China. *PLOS ONE* **12**, e0187405 (2017).
53. B. Cerasetti, L. Rouse, R. Arciero, T. Billings, M. Carra, A. Curci, J. de Grossi Mazzorin, L. Forni, É. Luneau, A. Potenza, R. N. Spengler, "The rise and decline of the desert cities: The last stages of the BMAC at Togolok 1" in *Cultures in Contact*, C. Baumer, M. Novák, S. Rutishauser, Eds. (Harrassowitz Verlag, Wiesbaden, 2022), pp. 89–116.
54. S. Salvatori, M. Tosi, Eds., *The Bronze Age and Early Iron Age in the Margian Lowlands: Facts and Methodological Proposals for a Redefinition of the Research Strategies. Archaeological Map of the Murghab Delta Studies and Reports, vol. II. BAR International Series 1806* (Archaeopress, Oxford, 2008).
55. B. Lyonnet, N. A. Dubova, "Questioning the Oxus Civilization or Bactria-Margiana Archaeological Culture (BMAC): An overview" in *The World of the Oxus Civilization* (Routledge, London, 2020), pp. 7–65.
56. Y. Zhang, P. Zhao, Z. Dong, D. Wang, P. Guo, X. Guo, Q. Song, W. Zhang, Q. Xia, Comparative proteome analysis of multi-layer cocoon of the silkworm *Bombyx mori*. *PLOS ONE* **10**, e0123403 (2015).
57. A. Fahn, E. Werker, P. Baas, Eds., *Wood Anatomy and Identification of Trees and Shrubs from Israel and Adjacent Regions* (Israel Academy of Sciences and Humanities, Jerusalem, 1986).
58. J. Q. Cheng, J. J. Yang, P. Liu, Eds., *Atlas of Wood in China* (China Forestry Publishing House, Beijing, 1992).
59. J. Li, Ed., *The Prehistoric Remains of His-yin Ts'un, A Preliminary Report* (Tsinghua University Research Institute, Beijing, 1927).
60. D. Kuhn, "The silk workshops of the Shang Dynasty (16th–11th Century BC)" in *Explorations in the History of Science and Technology in China*, D. Hu, Ed. (Shanghai Classics Publishing House, Shanghai, 1982), pp. 367–408.
61. E. J. W. Barber, Ed., *Prehistoric Textiles* (Princeton Univ. Press, Princeton, NJ, 1991).
62. H. L. Zhang, H. Y. Gao, Observation and analysis on the silk materials from Qingtai site, Xingyang. *Cult. Relics Cent. China* **3**, 10–16 (1999).
63. K. M. Zhou, The revelation on the silk remains from the Qianshanyang site. *Cult. Relics* **1**, 74–77 (1980).
64. H. Xu, Q. M. Ou, M. S. Li, H. Z. Zhang, The test on the silk materials from Qianshanyang site. *Silk Mon.* **2**, 43–45 (1981).
65. Y. Zhou, The origin of silk—The Qianshanyang site from Huzhou. *Silk Mon.* **6**, 1–5 (2006).
66. Zhejiang Provincial Institute of Cultural Relics and Archaeology, Ningbo Municipal Institute of Cultural Heritage Management, The Hemudu Site Museum of Yuyao City, *Excavation Report of the Neolithic Site—Hemudu* (Cultural Relics Press, Beijing, 2003).
67. The Shanxi Archaeological Team, Institute of Archaeology, Academic Sinica, Excavations of the Neolithic sites at Dongzhuang Cun and Xiwang Cun in Ruicheng County, Shanxi Province. *Acta Archaeol. Sin.* **1**, 1–63 (1973).
68. School of Archaeology Jilin University, Shanxi Provincial Academy of Archaeology, Yuncheng Cultural Relics Conservation Center, Excavation during 2019–2020 of the Neolithic site at Shicun in Xiaxian, Shanxi. *World Antiq.* **2**, 2–7 (2021).
69. K. Riboud, "A closer view of early Chinese silks" in *Studies in Early Textiles*, M. Gervers, Ed. (Univ. Toronto Press, Toronto, 1977), pp. 89–98.
70. Y. Zhou, L. L. Jia, J. Liu, Scientific research on the textiles excavated from a Zoroastrian cemetery in Pamir. *Sci. Conserv. Archaeol.* **31**, 55–64 (2019).
71. P. G. Bahn, Ed., *The Atlas of World Geology* (Checkmark Books, New York, 2000), p. 128.
72. A. N. Gulati, "A note on the early history of silk in India" in *Technical Reports on Archaeological Remains III*, J. Clutton-Brock, Vishnu-Mittre, A. N. Gulati, Eds. (Deccan College, Pune, 1961), pp. 53–59.
73. E. Panagiotakopulu, P. C. Buckland, P. M. Day, C. Doumas, A. Sarpaki, P. Skidmore, A lepidopterous cocoon from Thera and evidence for silk in the Aegean Bronze Age. *Antiquity* **71**, 420–429 (1997).

74. C. H. Greenewalt, N. Cahill, H. Dedeoğlu, P. Herrmann, W. Rast, "The Sardis Campaign of 1986" in *Preliminary Reports of ASOR-Sponsored Excavations* (The John Hopkins University Press, 1990), pp. 137–177.
75. H.-J. Hundt, Über vorgeschichtliche Seidenfunde. *Jahrb. Römisch-Ger. Zent. Mus.* **16**, 59–71 (1970).
76. I. Good, "When east met west: Interpretative problems in assessing Eurasian contact and exchange in antiquity" in *New Directions in Silk Road Archaeology. Proceedings of a Workshop*, A. V. G. Betts, F. Kidd, Eds. (Dietrich Reimer, Berlin, 2010), pp. 35–48.
77. I. Good, "Strands of connectivity: Assessing the evidence for long distance exchange of silk in Later Prehistoric Eurasia" in *Interweaving Worlds: Systemic Interactions in Eurasia, 7th–1st Millennia BC*, T. C. Wilkinson, S. Sherratt, J. Bennet, Eds. (Oxbow, Oxford, 2011), pp. 218–230.
78. G. Riek, H.-J. Hundt, Eds., *Der Hohmichele: Ein Fürstengrabhügel der späten Hallstattzeit bei der Heuneburg* (Römisch-Germanische Forschungen 25, de Gruyter, Berlin, 1962).
79. J. Banck-Burgess, "Die Textilfunde aus dem hallstattzeitlichen Fürstengrab von Hochdorf, Gemeinde Eberdingen (Kreis Ludwigsburg)" in *NESAT V. Textilsymposium Neumünster: Archäologische Textilfunde, 4–7.5.1993*, G. Jaacks, K. Tidow, Eds. (Textilmuseum Neumünster, Neumünster, 1994), pp. 43–52.
80. J. Banck-Burgess, Ed., *Hochdorf IV, Die Textilfunde aus dem späthallstattzeitlichen Fürstengrab von Eberdingen-Hochdorf (Kreis Ludwigsburg) und weitere Grabtextilien aus hallstatt-latènezeitlichen Kulturgruppen* (Forschungen und Berichte zur Vor- und Frühgeschichte 70, Konrad Theiss, Stuttgart, 1999).
81. J. Banck-Burgess, "Case study: The textiles from the princely burial at Eberdingen-Hochdorf, Germany" in *Textiles and Textile Production in Europe: From Prehistory to AD 400*, M. Gleba, U. Mannering, Eds. (Oxbow, Oxford, 2012), pp. 139–150.
82. K. Mann, "Aminosäurenanalysen" in *Hochdorf IV, Die Textilfunde aus dem späthallstattzeitlichen Fürstengrab von Eberdingen-Hochdorf (Kreis Ludwigsburg) und weitere Grabtextilien aus hallstatt-latènezeitlichen Kulturgruppen*, J. Banck-Burgess, Ed. (Forschungen und Berichte zur Vor- und Frühgeschichte 70, Konrad Theiss, Stuttgart, 1999), pp. 235–238.
83. H.-J. Hundt, "Die Textilien im Grab von Hochdorf" in *Der Keltenfürst von Hochdorf. Methoden und Ergebnisse der Landesarchäologie, Katalog zur Ausstellung Stuttgart, Kunstgebäude vom 14 August bis 13 Oktober 1985*, D. Planck, J. Biel, G. Süsskind, A. Wais, Eds. (Landesdenkmalamt Baden-Württemberg, Stuttgart, 1985), pp. 106–115.
84. H. Heyart, Textilreste. *Hémecht* **24**, 500–501 (1972).
85. G. Thill, "Altrier" in *Trésors des princes celtes*, J.-P. Mohen, A. Duval, C. Eluère, Eds. (Réunion des musées nationaux, Paris, 1987), pp. 251–254.
86. B. Cerasetti, R. Arciero, M. Carra, A. Curci, J. de Grossi Mazzorin, L. Forni, É. Luneau, L. M. Rouse, R. N. Spengler, "Bronze and Iron Age urbanization in Turkmenistan: Preliminary results from the excavation of Togolok 1" in *Urban Cultures of Central Asia from the Bronze Age to the Karakhanids*, C. Baumer, M. Novák, Eds. (Harrassowitz Verlag, Wiesbaden, 2019), pp. 63–72.
87. E. Luneau, Ed., *La Fin de la Civilisation de l'Oxus* (de Boccard, Paris, 2014).
88. S. Salvatori, "Thinking around Grave 3245 in the 'royal graveyard' of Gonur" in *Na Puti Otkrytija Tsivilizatsii*, P. M. Kozhin, M. F. Kosarev, N. A. Dubova, Eds. (Aletejja, St. Petersburg, 2010), vol. 3, pp. 244–257.
89. V. I. Sarianidi, N. A. Dubova, T. J. Vallée-Raevsky, Nouvelles sépultures sur le territoire de la "nécropole royale" de Gonur Dépé. *Arts Asiat.* **65**, 5–26 (2010).
90. N. A. Dubova, "The 'royal necropolis' at Gonur Depe" in *The World of the Oxus Civilization* (Routledge, London, 2020), pp. 334–364.
91. V. I. Sarianidi, "Drevnjaja Baktrija: Novye aspekty staroj problem" in *Etnicheskie Problemy Istorii Tsentral'noj Azii v Drevnosti* (Nauka, Moscow, 1981), pp. 180–191.
92. V. I. Sarianidi, "Ob odnoj gruppe drevnebaktrijskoj gliptiki" in *Drevnjaja Indija i Nauka* (Nauka, Moscow, 1982), pp. 297–306.
93. K. Kaniuth, Long distance imports in the Bronze Age of Southern Central Asia. *Archäol. Mitt. Iran Turan* **42**, 3–22 (2010).
94. V. I. Sarianidi, Ed., *Margush: Mystery and True of the Great Culture* (Türkmendöwlethabarlary, Ashgabat, 2008).
95. D. Frenez, Manufacturing and trade of Asian elephant ivory in Bronze Age Middle Asia: Evidence from Gonur Depe. *Archaeol. Res. Asia* **15**, 13–33 (2017).
96. D. Frenez, "Torgovlja slonovoj kost'ju iz bivnej Asiatskogo slona" in *Trudy Margianskoj Arkheologicheskoj Ekspeditsii*, N. A. Dubova, Ed. (Staryj Sad, Moscow, 2018), vol. 7, pp. 106–133.
97. V. I. Sarianidi, Ed., *Gonurdepe, Türkmenistan: City of Kings and Gods* (Miras, Ashgabat, 2005).
98. V. I. Sarianidi, N. G. O. Boroffka, N. A. Dubova, Cultural contacts of Margiana, Turkmenistan, in the 3rd millennium BC: New evidence from Gonur Depe, Burial 4150. *Gandhāran Studies* **6**, 1–17 (2012).
99. V. I. Sarianidi, N. G. O. Boroffka, N. A. Dubova, "Kul'turnye kontakty Margiany v III tys. do n.e." in *Trudy Margianskoj Arkheologicheskoj Ekspeditsii*, V. I. Sarianidi, Ed. (Staryj Sad, Moscow, 2014), vol. 4, pp. 127–137.
100. R. N. Spengler, I. de Nigris, B. Cerasetti, M. Carra, L. M. Rouse, The breadth of dietary economy in Bronze Age Central Asia. *J. Archaeol. Sci. Rep.* **22**, 372–381 (2018).
101. B. Lyonnet, N. A. Dubova, Eds., *The World of the Oxus Civilization* (Routledge, London, 2020).
102. R. M. Sataev, N. A. Dubova, M. A. Mamedov, "The internal chronology and periodization of Gonur Depe" in *Antiquities of East Europe, South Asia and South Siberia in the Context of Connections and Interactions within the Eurasian Cultural Space (New Data and Concepts): Proceedings of the International Conference* (IIMK RAS, 2019), 41 pp.
103. O. Lecomte, "Ulug-depe: 4000 years of evolution between plain and desert" in *Historical and Cultural Sites of Turkmenistan* (Turkmen State Publishing Service, Ashgabat, 2011), pp. 223.
104. R. Besenval, A. I. Isakov, Sarazm et les débuts du peuplement agricole dans la région de Samarkand. *Arts Asiat.* **44**, 5–20 (1989).
105. P. L. Kohl, C. C. Lamberg-Karlovsky, R. Maddin, Metallurgical analysis from Sarazm, Tadjikistan SSR. *Archaeometry* **29**, 90–102 (1987).
106. F. T. Hiebert, K. Kurbansakhatov, Eds., *A Central Asian Village at the Dawn of Civilization: Excavations at Anau, Turkmenistan* (Univ. Pennsylvania Museum of Archaeology and Anthropology, Philadelphia, PA, 2003).
107. P. L. Kohl, The Namazga civilization: An overview. *Sov. Anthropol. Archaeol.* **19**, vii–xxxviii (1981).
108. E. E. Kuzmina, *The Origin of the Indo-Iranians*, J. P. Mallory, Ed. (Brill, Leiden, 2007).
109. A. I. Isakov, Sarazm: An agricultural center of ancient Sogdiana. *Bull. Asia Inst.* **8**, 1–12 (1994).

Acknowledgments: We are grateful to our research partners from the Institute of Archaeology of the Uzbek Academy of Sciences (Samarkand) for assistance within fieldwork. We also thank H. Wang and L. Lin from the Institute of Vertebrate Paleontology and Paleoanthropology, Chinese Academy of Sciences, for assistance with the laboratory work. **Funding:** This work was supported by the following: National Key Research and Development Program of China grant nos. 2022YFF0801102 and 2022YFE0203800 (to X.Z.), Chinese Academy of Sciences Project for Young Scientists in Basic Research grant no. YSBR-019 (to X.Z.), European Research Council (ERC) grant no. 851102 (to R.N.S.), The Youth Innovation Promotion Association of Chinese Academy of Sciences grant no. 2022071 (to H.S.), and National Key Research and Development Program of China grant no. 2022YFD1201600 (to Q.X.). **Author contributions:** Conceptualization: X.Z., H.S., R.N.S., M.K., J.M., J.W., X.Li., A.A.A., Y.W., X.Liu. Data curation: X.Z., H.S., and X.Li. Formal analysis: X.Z., H.S., and Y.W. Funding acquisition: X.Z., H.S., Q.X., and X.Li. Investigation: X.Z., H.S., M.K., J.W., A.A.A., F.M., H.R., K.Z., X.Li., and X.Liu. Methodology: X.Z., H.S., Z.L., H.R., M.K., J.W., X.Li., and K.Z. Project administration: X.Z., H.S., M.K., J.M., J.W., and X.Li. Resources: X.Z., M.K., J.M., J.W., A.A.A., F.M., and X.Li. Software: H.S. Supervision: X.Z., M.K., J.M., J.W., A.A.A., F.M., X.Li., and X.Liu. Validation: X.Z., H.S., G.C., H.R., M.K., and A.A.A. Visualization: X.Z., H.S., G.C., J.W., and X.Li. Writing—original draft: X.Z., H.S., R.N.S., X.Li., and X.Liu. Writing—review and editing: X.Z., H.S., Z.L., R.N.S., Q.X., J.M., J.W., A.A.A., X.Li., and X.Liu. **Competing interests:** The authors declare that they have no competing interests. **Data, code, and materials availability:** All data and code needed to evaluate and reproduce the results in the paper are present in the paper and/or the Supplementary Materials. This study did not generate new materials.

Submitted 6 October 2025
Accepted 16 June 2026
Published 23 July 2026
10.1126/sciadv.aec8738
